# Multipole Vortex Blobs (MVB): Symplectic Geometry and Dynamics

**DOI:** 10.1007/s00332-017-9367-4

**Published:** 2017-03-16

**Authors:** Darryl D. Holm, Henry O. Jacobs

**Affiliations:** Department of Mathematics, Imperial College, London, SW7 2AZ UK

**Keywords:** Vortex blob methods, Singular momentum maps, Regularized Euler fluid equations, Hamiltonian dynamics, 76M23, 76M60, 70H15

## Abstract

Vortex blob methods are typically characterized by a regularization length scale, below which the dynamics are trivial for isolated blobs. In this article, we observe that the dynamics need not be trivial if one is willing to consider distributional derivatives of Dirac delta functionals as valid vorticity distributions. More specifically, a new singular vortex theory is presented for regularized Euler fluid equations of ideal incompressible flow in the plane. We determine the conditions under which such regularized Euler fluid equations may admit vorticity singularities which are stronger than delta functions, e.g., derivatives of delta functions. We also describe the symplectic geometry associated with these augmented vortex structures, and we characterize the dynamics as Hamiltonian. Applications to the design of numerical methods similar to vortex blob methods are also discussed. Such findings illuminate the rich dynamics which occur below the regularization length scale and enlighten our perspective on the potential for regularized fluid models to capture multiscale phenomena.

## Introduction

Vortices are important in hydrodynamics because they are the sources for the incompressible flow field. The vorticity distribution at any instant of time determines both the current state of the flow and its future evolution, for given boundary conditions. This property holds for any Hamiltonian system, and it can indeed be shown that the dynamics of vortices can be usefully expressed in Hamiltonian form. In the vorticity and stream function formulation of an ideal incompressible planar fluid, the evolution of the vorticity distribution $\omega (x,y,t)$ is given by

1 $$\begin{aligned} \partial _t \omega - \{\omega ,\psi \} \equiv \partial _t \omega - \partial _x \omega \, \partial _y \psi + \partial _y \omega \, \partial _x \psi = 0\,, \end{aligned}$$

where $\omega = - \Delta \psi$ is the vorticity, $\psi$ is the stream function, and $\Delta = \partial _{xx} + \partial _{yy}$ is the Laplace operator. The corresponding (*x*, *y*) components of the Eulerian velocity field are given by

$$\begin{aligned} (u,v) = (\partial _y \psi , - \partial _x \psi ). \end{aligned}$$

If one is willing to view the vorticity $\omega$ as a distribution, one can consider point vortex solutions. In particular, point vortices are obtained if one considers the vorticity solution ansatz

$$\begin{aligned} \omega (z,t) = \sum _{i} \Gamma _i(t) \delta _{z_i(t)}\,, \end{aligned}$$

where $\Gamma _i(t) \in {\mathbb {R}}$, $z=(x,y) \in {\mathbb {R}}^2$ and $\delta _{z_i(t)}$ is the Dirac delta distribution centered at the point $z_i(t) = (x_i(t),y_i(t)) \in {\mathbb {R}}^2$ at a given time $t \in {\mathbb {R}}$. Substitution of this ansatz into (1) yields the following well-known finite dimensional system in the form of Hamilton’s canonical equations,

2 $$\begin{aligned} \begin{aligned} \frac{\mathrm{d} \Gamma _i}{\mathrm{d}t}&= 0, \quad \psi (z,t) = \sum _i \Gamma _i(t) G(z-z_i(t)), \\ \frac{\mathrm{d}x_i}{\mathrm{d}t}&= \partial _y \psi (z_i),\quad \frac{\mathrm{d}y_i}{\mathrm{d}t} = - \partial _x \psi (z_i), \end{aligned} \end{aligned}$$

where $G(z) = - (2\pi )^{-1} \ln ( \Vert z \Vert )$ is the Green’s function for the planar Laplacian.

A point vortex approximation to a continuous distribution of vorticity for Euler’s fluid equations is problematic, though a point vortex induces a flow velocity which becomes unbounded. However, when the point vortex is made smooth and bounded (regularized), the approximation becomes reasonable (Chorin 1973).

For example, one may consider the regularized form of the vorticity equation given by choosing a translationally and rotationally invariant smoothing kernel $K_\delta$ of width $\delta > 0$ and defining the regularized vorticity as $K_\delta * \omega = -\Delta \psi$ while continuing to use (1) to evolve $\omega$ in time. For example, $K_\delta (z) = \exp ( - \Vert z\Vert ^2 / \delta ^2)$ is considered in Beale and Majda (1985). In this case, the point vortex ansatz yields (2) again, except that the singular Green’s function *G* is replaced by the smooth kernel

3 $$\begin{aligned} G_\delta (z) := K_\delta *G(z) = \frac{1}{4\pi } \left( \mathrm{Ei}(- \Vert z \Vert ^2 / \delta ^2) - 2\ln ( \Vert z \Vert ) \right) , \end{aligned}$$

where $\mathrm{Ei}( \cdot )$ denotes the exponential integral function. The vorticity kernel $G_\delta$ has no singularity at the origin for $\delta > 0$ and is known as a *vortex blob*. This system is the starting point for the vortex blob method, introduced in Chorin (1973) (albeit with a different regularization).

The economy of the vortex blob method derives from the property that Dirac delta distributions are hyper-local (i.e., parametrized by position), and the property that the vorticity equation (1) admits Dirac delta distributions as solutions. However, there are many distributions which are localized to a similar degree (e.g., derivatives of delta functions, $\partial _x \delta _{z_i}$).

In this paper, we study the more general vorticity solution ansatz,

$$\begin{aligned} \omega (z,t) = \sum _{i,m,n} \Gamma _i^{mn}(t) \partial _x^m \partial _y^n \delta _{z_i}\,. \end{aligned}$$

We find that this ansatz yields a closed finite dimensional system which generalizes vortex blobs. We call these new carriers of vorticity *multipole vortex blobs* or *MVBs*.

### Main Contributions

1. Section 2 briefly reviews the background for vortex methods in fluid modeling.
2. Section 3 reviews the relationship between regularized fluids and vortex blob methods.
3. Section 4 derives the equations of motion for point vortices and MVBs as exact solutions of a regularized vorticity equation.
4. Section 5 derives the conservation laws for these equations, such as energy, linear momentum, and angular momentum, and circulation. The derivation of these conserved quantities as symplectic momentum maps can be found in Appendix B.
5. Section 6 explains the relationship between the dynamical systems for MVBs and an implicitly defined *closed* dynamical system which governs the spatial moments of the vorticity distribution.
6. Section 7 discusses numerical aspects of using MVBs to model fluid dynamics, such as approximations of initial conditions (Sect. 7.2), and grouping of computational nodes (Sect. 7.1).
7. Section 8 presents the results of several numerical experiments involving small numbers of vortices, for $N=1,2$, and 3.
8. MVB dynamics are Hamiltonian. We present the symplectic and Hamiltonian structure of MVB dynamics in Sect. 9.

## Background

Vortex methods for fluid modeling predate the computer age, and references to them can be found in the work of Helmholtz (Smith 2011, see the introductory section). For example, the use of point vortices as idealized solutions can already be found in a 1931 paper concerning a “line of discontinuity” in planar fluid flow (Rosenhead 1931). At the beginning of their development, the infinite velocities (and energies) associated with point vortices caused great difficulties, both numerically and theoretically. In fact, the point vortex approach did not produce a competitive numerical method until the 1970s, when the problems related to singularities were overcome by regularizing the singular vortex kernel to form a *vortex blob*. Stochastic perturbations were further included to model viscosity (Chorin 1973). These adjustments to the classical point vortex method yielded the *vortex blob method*, which quickly became of practical use for realistic fluid flow modeling. In particular, the regularized system proved more amenable to error analysis. It was shown that the solutions of the vortex blob method converge to solutions of the Navier–Stokes equations in Hald (1979). Later, stronger convergence rates were achieved by judicious choice of vortex kernels. By convolving the singular vortex kernel with sums of Gaussian smoothing kernels, a sequence of vortex blob kernels with faster convergence rates was found. Specifically, the convergence rate of the *m*th kernel was found to be of order $h^{mq}$ for any $q \in (0,1)$ where $h = \delta ^q$ is a grid-spacing parameter and $\delta > 0$ is a length scale associated with the smoothing kernel (Beale and Majda 1982, 1985).

Simultaneously, the symplectic geometry of point vortices was clarified in Marsden and Weinstein (1983) by invoking Arnold’s interpretation of ideal fluids (Arnold 1966). The findings of Marsden and Weinstein (1983) were developed further in Gay-Balmaz and Vizman (2012) to handle fluid flow on manifolds with nontrivial homology. While this theoretical development clarified the geometry of point vortices, vortex blobs were sometimes thought to be a numerical “trick” which violated the geometric interpretation. However, this thought was banished with the invention of the Euler-$\alpha$ model, a regularized model of ideal fluids with a parameter $\alpha$ representing the typical correlation length of fluctuations away from the mean of a Lagrangian fluid path (Foias et al. 2001). In particular, vortex blob solutions associated with a specific kernel serve as *exact* solutions to the Euler-$\alpha$ model (Oliver and Shkoller 2001). The Euler-$\alpha$ kernel is different from the kernels used in Chorin (1973) and Beale and Majda (1985). A comparison of the Euler-$\alpha$ kernel to the $m=1$ kernel of Beale and Majda (1985) is given in Holm et al. (2006) for vortex filament and vortex sheet motion.

While vortex blobs performed well, they did not capture all of the qualitative richness observed in fluid vorticity dynamics. In particular, blobs of vorticity in real ideal fluids are known to change shape and deviate from initially circular distributions. A numerical method is proposed in Rossi (1997, 2005) to capture these shape dynamics by adding basis functions with nontrivial moments in the study of vortex merger (see, for example, Melander et al. 1988; Le Dizès and Verga 2002; Meunier et al. 2005). Another distinct model obtained by projection onto a Hermite basis is described in Nagem et al. (2009). This projection yielded a finite dimensional systems which modeled the (truncated) moments of the vorticity of an ideal incompressible fluid. The derivation of simplified combinatorial formulas invoked by the dynamics of this model was discovered in Uminsky et al. (2012), and these formulas have made the method numerically tractable for a large number of moments.

A dual approach to the moment-based methods of the previous paragraph (Rossi 1997, 2005; Nagem et al. 2009) is to consider multipole-based methods. This is the approach proposed in Nicolaides (1986), where an initial vortex ansatz consisting of sums of distributional derivatives of dirac delta distributions is considered. Such an idea has occurred intermittently in various forms in the literature, over many years. For example, a regularized vortex blob model, in the spirit of Beale and Majda (1982, 1985), which considered vorticity distributions of the form $\omega = \sum \Gamma _i \delta _{z_i} + \Gamma _i^x \partial _x \delta _{z_i} + \Gamma _i^y \partial _y \delta _{z_i}$ was investigated in Chiu and Nicolaides (1988). Here it was proven that this augmentation of the traditional vortex method will yield faster spectral convergence than that of a traditional vortex blob method. The current article considers higher order derivatives and can be seen as a natural follow-up to (Chiu and Nicolaides 1988). More recently, dynamics have been derived for interactions of pure vortices and pure dipoles. These come from vorticity distributions of the form $\omega = \sum _{i} \Gamma _i \delta _{z_{v,i}} + \sum _{j} \left( \Gamma _i^x \partial _x \delta _{z_{d,i}} + \Gamma _i^y \partial _y \delta _{z_{d,i}}\right)$ with the assumption that the locations of the dipoles, and the vortices never overlap and that their self-interaction terms may be neglected (Yanovsky et al. 2009; Tur et al. 2011). In a different approach, approximations of dipoles are created by holonomically constraining vortices of opposite strength to be a fixed distance from one another (Tchieu et al. 2012). The question remains, however, to what extent the dynamics of Tchieu et al. (2012) approximates those of Yanovsky et al. (2009) and Tur et al. (2011) after self-interaction terms have been neglected. In summary, the removal of self-interaction terms is one of the primary obstacles to obtaining a multipole-based generalization of the point vortex method (Smith 2011). Moreover, the spectral error decay rates found in Hald (1979), Beale and Majda (1982, 1985) and Chiu and Nicolaides (1988) arise from the use of vortex blobs in place of (singular) point vortices. In this article, we will follow this regularization-based approach.

## Vortex Blobs and Regularized Fluid Models

In this section, we review a class of regularized fluid models and their relationship with vortex blob methods (for a more detailed discussion see Holm et al. 2006). The sort of fluid models we consider take the form

$$\begin{aligned} \partial _{t} \omega + \mathbf {u} \cdot \nabla \omega = 0 \\ \omega = \mathrm{curl}( L_{\alpha } \cdot u ). \end{aligned}$$

where $L_{\alpha }$ is a ${\text {SE}}(2)$ invariant linear psuedo-differential operator with a length scale parameter $\alpha > 0$ such that $\lim _{\alpha \rightarrow 0} Q_{op} = 1$. When $L_{\alpha }$ is just the identity, the above “model” is Euler’s equations of motion for an ideal fluid. When $L_{\alpha } = (1- \alpha ^{-2} \Delta )$ where $\Delta$ is the Laplace operator, then we obtain the the Euler-$\alpha$ model, the solutions of which will converge to solutions of Euler’s ideal fluid equations as $\alpha >0$ vanishes (Foias et al. 2001).

We may replace *u* with its stream function, $\psi$, in order to rewrite the above equations as

4 $$\begin{aligned} \partial _{t} \omega + \{ \psi , \omega \} = 0 \end{aligned}$$

5 $$\begin{aligned} \omega = \Delta ( L_{\alpha } \cdot \psi ) \end{aligned}$$

This allows us to represent planar fluid dynamics in terms of scalar functions and distributions rather than vector fields.

The relationship between these regularized models and vortex blobs methods comes from first considering the point vortex ansatz

$$\begin{aligned} \omega (z ; t) = \sum _{i} \Gamma _{i} \delta (z - z_{i}(t) ). \end{aligned}$$

If the operator, $\Delta \circ L_\alpha$, has a non-singular Green’s function, $G_\alpha$, then substituting the ansatz into (5) implies that

6 $$\begin{aligned} \psi (z;t) = \sum _{i} \Gamma _{i} G_{\alpha }(z - z_{i}(t)) \end{aligned}$$

We should note that when $L_\alpha$ is the identity (i.e., for an Euler fluid), then $G_\alpha$ is singular, and an extra argument (perhaps a physical one) must be presented in order to allow the resulting singular velocity fields. In this paper, no such issues with singularity arise because we are modeling an Euler fluid with a regularized fluid where $L_{\alpha }$ has a non-singular Green’s function.

Substitution of $\psi$ into (4) then implies the following equations of motion for the vortex cores $z_{i} = (x_{i},y_{i})$ and the strengths $\Gamma _{i}(t)$:

7 $$\begin{aligned} \frac{\mathrm{d}x_{i}}{\mathrm{d}t} = \partial _{y}\psi (z_{i}(t);t), \quad \frac{\mathrm{d}y_{i}}{\mathrm{d}t} = - \partial _{x} \psi (z_{i}(t);t ),\quad \frac{\mathrm{d}\Gamma _{i}}{\mathrm{d}t} = 0. \end{aligned}$$

When $\alpha = 0$ and $L_{\alpha } = 1$, this is nothing but the point vortex method. When $\alpha > 0$, it is possible for $\psi$ to be much more regular, and we obtain various vortex blob methods. In particular, we obtain the smooth vortex blobs of Beale and Majda (1985).

It is notable that (9) and (7) together form a finite dimensional ODE. The solutions of this ODE are *exact* solutions to the regularized fluid model. Again, this is valuable because the solutions of many regularized fluid models have been shown to converge to solutions of the ideal fluid equations as $\alpha$ vanishes. This paper seeks to generalize these point-like solutions to regularized fluid models to obtain a richer class of solutions with richer conservation properties.

## Equations of Motion

In this section, we derive the equations of motion for the time-dependent parameters which specify multipole vortex blobs (MVBs). The zeroth-order MVBs are just standard vortex blobs, and the resulting equations of motion are those of the standard (non-stochastic) vortex blob algorithm (Chorin 1973). The first-order MVBs are regularized dipoles, and the equations of motion are those of Chiu and Nicolaides (1988). Here we will derive the equations of motion for *N*th-order MVBs following the approach of Chiu and Nicolaides (1988).

Consider the ansatz for the vorticity,

8 $$\begin{aligned} \omega (z,t) = \sum _{i \in S} \sum _{m+n \le N} \Gamma ^{mn}_i(t) \partial _x^m \partial _y^n \delta _{z_i} \,, \end{aligned}$$

for spatially constant dynamical variables $\Gamma ^{mn}_i(t) \in {\mathbb {R}}$ for $i \in S$ where *S* is some countable set. The stream function is

9 $$\begin{aligned} \psi (z,t) = \sum _{i \in S} \sum _{m+n \le N} \Gamma ^{mn}_i(t) \partial _x^m \partial _y^n G_\delta (z-z_i(t) ) \,. \end{aligned}$$

The corresponding velocity field is given by

10 $$\begin{aligned} {\left\{ \begin{array}{ll} u(z,t) = \partial _y \psi (z,t) = \sum _{i \in S,m+n \le N} \Gamma ^{mn}_i(t) \partial _x^m \partial _y^{n+1} G_\delta (z-z_i(t) ) \,, \\ v(z,t) = -\partial _x \psi (z,t) = - \sum _{i \in S, m+n \le N} \Gamma ^{mn}_i(t) \partial _x^{m+1} \partial _y^n G_\delta (z-z_i(t) )\,. \end{array}\right. } \end{aligned}$$

**Fig. 1:**
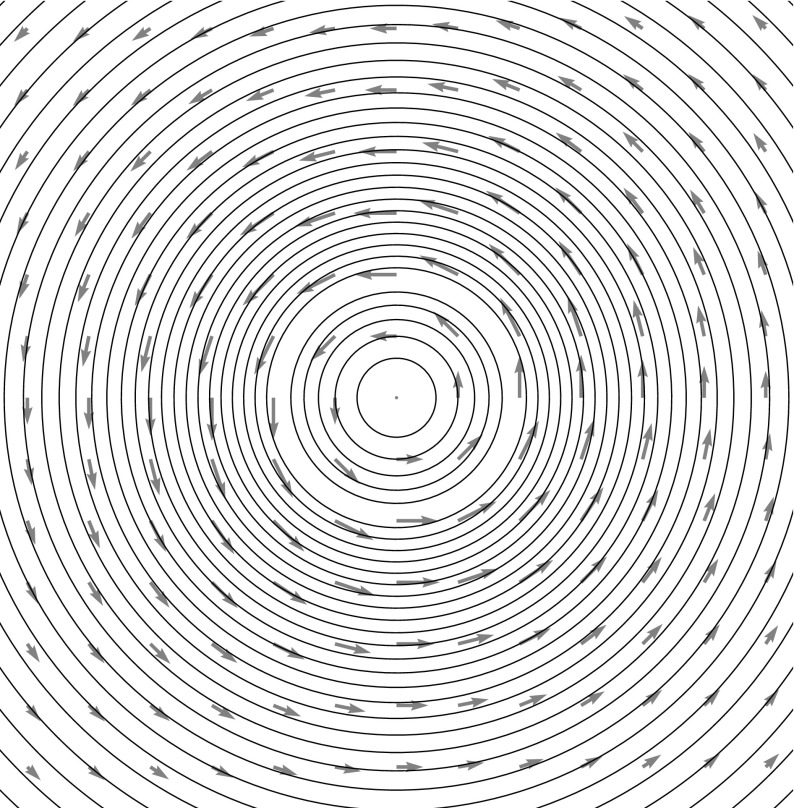
A zeroth-order MVB with $z = 0$ and $\Gamma =1$, using the kernel $G_\delta$ of Eq. (3). This form of the kernel produces one of the vortex blobs presented in Beale and Majda (1985), and the resulting numerical method is identical

Examples of the types of velocity fields produced are depicted in Figs. 1 through 3 on page 7.

**Fig. 2:**
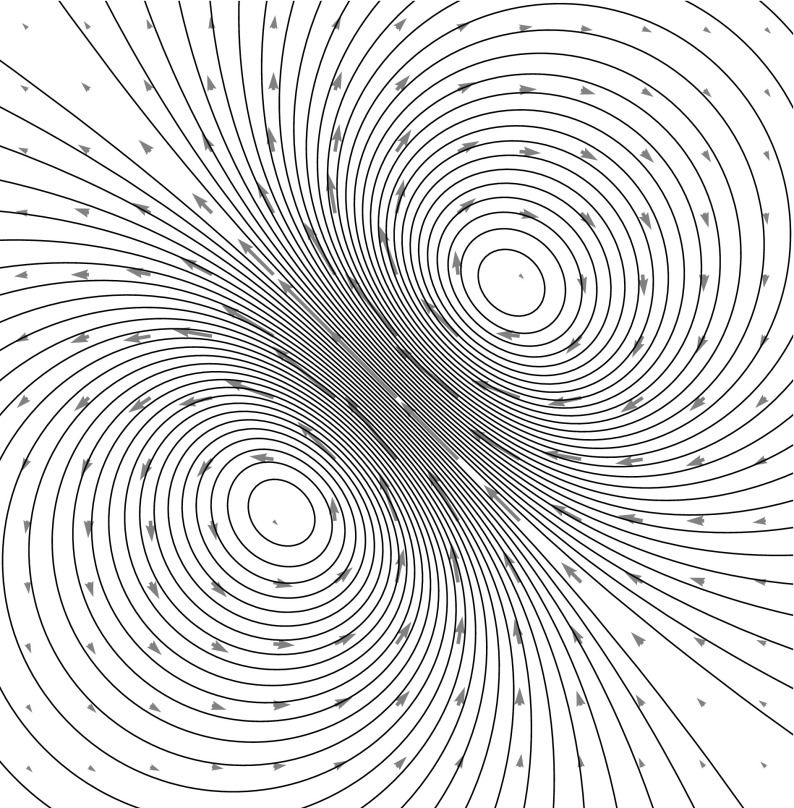
The flow field around a first-order MVB with $\Gamma = 0,\Gamma ^x = 1,\Gamma ^y=1$ is that of a regularized dipole

**Fig. 3:**
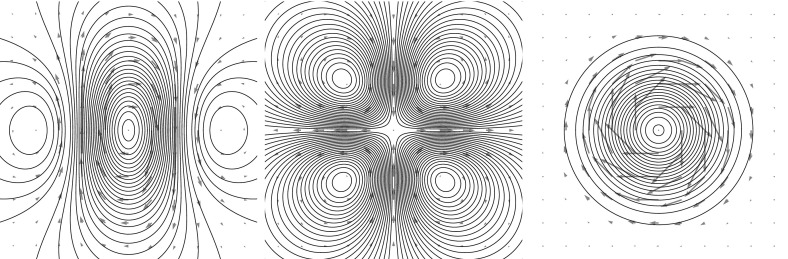
Various second-order MVBs with all $\Gamma$’s set to zero except. (*left*) $\Gamma ^{xx} = 1$, (*middle*) $\Gamma ^{xy}=1$, (*right*) $\Gamma ^{xx}=\Gamma ^{yy}=1$

We seek equations of motion for the $\Gamma ^{mn}_i(t)$’s and $z_i(t)$’s such that the velocity field (10) satisfies the vorticity equation (1). In the following calculations, we will not show the explicit time dependence of the dynamical variables.

We now find

$$\begin{aligned} \partial _t \omega = \sum _{ \begin{array}{c} i \in S \\ m+n \le N \end{array}} \frac{\mathrm{d} \Gamma _i^{mn}}{\mathrm{d}t} \partial _x^m \partial _y^n \delta _{z_i} - \Gamma _i^{mn} \frac{\mathrm{d}x_i}{\mathrm{d}t} \partial _{x}^{m+1} \partial _y^{n} \delta _{z_i} - \Gamma _i^{mn} \frac{\mathrm{d}y_i}{\mathrm{d}t} \partial _{x}^{m} \partial _y^{n+1} \delta _{z_i}, \end{aligned}$$

and

$$\begin{aligned} \partial _y \psi \, \partial _x \omega = \sum _{ \begin{array}{c} i \in S \\ m+n \le N \end{array}} \partial _y \psi \, \Gamma _i ^{mn}\partial _x^{m+1} \partial _y^n \delta _{z_i}\,. \end{aligned}$$

By invoking (24) of Appendix 1, we can rearrange the previous equation to obtain

$$\begin{aligned} \partial _y \psi \, \partial _x \omega&= \sum _{ \begin{array}{c} i \in S \\ m+n \le N \\ \ell ,k \end{array}}\Gamma _i^{mn} (-1)^{m+n+1 + \ell + k} \left( {\begin{array}{c}m+1\\ \ell \end{array}}\right) \left( {\begin{array}{c}n\\ k\end{array}}\right) \partial _x^{\ell } \partial _y^{k+1} \psi (z_i) \partial _x^{m+1-\ell } \partial _y^{n-k} \delta _{z_i}. \end{aligned}$$

Similarly, we find

$$\begin{aligned} \partial _x \psi \, \partial _y \omega&= \sum _{\begin{array}{c} i \in S \\ m+n \le N\\ \ell ,k \end{array} } \Gamma _i^{mn} (-1)^{m+n+1 + \ell + k} \left( {\begin{array}{c}m\\ \ell \end{array}}\right) \left( {\begin{array}{c}n+1\\ k\end{array}}\right) \partial _x^{\ell +1} \partial _y^k \psi (z_i) \partial _x^{m-\ell } \partial _y^{n+1-k} \delta _{z_i}. \end{aligned}$$

Substitution of these expressions into (1) yields the vanishing of a linear combination of the distributions $\partial _x^m \partial _y^n \delta _{z_i}$ for $m+n \le N+1$. Since each of these distributions is linearly independent of the others (assuming the $z_i$’s are distinct), their individual coefficients must each vanish independently. If we focus on the terms of the sum where $m+n = N$, we obtain coefficients for $\partial _{x} \delta$ and $\partial _{y}\delta$ at the core locations. The vanishing of these coefficients yields the dynamics for MVB cores

11 $$\begin{aligned} \frac{\mathrm{d}x_i}{\mathrm{d}t} = \partial _y \psi (z_i), \quad \frac{\mathrm{d}y_i}{\mathrm{d}t} = - \partial _x \psi (z_i) . \end{aligned}$$

The vanishing of the coefficient of $\delta _{z_i}$ yields

$$\begin{aligned} \frac{\mathrm{d}\Gamma ^{0,0}_i}{\mathrm{d}t} = 0. \end{aligned}$$

For $\ell +k \le N$, the vanishing of the coefficient of $\partial _x^{\ell } \partial _{y}^{k} \delta _{z_i}$ yields

12 $$\begin{aligned} \begin{aligned}&\frac{\mathrm{d}\Gamma _i^{\ell k}}{\mathrm{d}t} \\&=(-1)^{\ell + k} \sum _{ \begin{array}{c} m> \ell \\ n > k \\ n+m \le N \end{array} }\Gamma _i^{mn} \Bigg [ \left( {\begin{array}{c}n\\ k\end{array}}\right) \left( {\begin{array}{c}m\\ \ell -1\end{array}}\right) + \left( {\begin{array}{c}n\\ k-1\end{array}}\right) \left( {\begin{array}{c}m\\ \ell \end{array}}\right) \Bigg ] \partial _{m-\ell +1,n-k+1} \psi (z_i) \end{aligned} \end{aligned}$$

We observe that $\mathrm{d} \Gamma _{i}^{\ell k}/\mathrm{d}t$ depends on $\psi$ at the vortex cores $z_{i}$, and the vortex core dynamics depend on $\psi$ as well. Fortunately, we already found that $\psi$ is purely a function of $\Gamma _{i}^{\ell k}$ and $z_{i}$, as stated in (9). Thus, (12) and (11) (with (9)) form a closed finite dimensional system. Most notably, by construction the vorticity equation (1) admits the *N*th-order MVB ansatz for the vorticity in (8) as a solution when the $z_i(t)$’s and the $\Gamma _i(t)$’s satisfy the just derived finite dimensional system.

### Remark 4.1

In the point vortex method (i.e., the unregularized case where $L_{\alpha } = 1$), this derivation of the dynamics requires an extra step. In particular, one must discard the self-interaction term, which we will describe here. For point vortices, $\omega = - \Delta \psi$. Substituting the point vortex ansatz $\omega = \sum _i \Gamma _i \delta (z - z_i(t))$ into the equations of motion (4) would then yield the nonsensical equation

$$\begin{aligned} {\dot{z}}_i = \nabla ^\perp \left( \sum _{j} \Gamma _j \log | z_i - z_j | \right) . \end{aligned}$$

We say “non-sensical,” because the right-hand side explodes when you evaluate the *i*th term in the sum, the self-interaction term. Historically, it is customary to discard this self-interaction term based on physical and symmetry principles (Marchioro and Pulvirenti 1994, Chapter 4). In contrast, for blob methods the logarithmic kernel is replaced with a differentiable kernel function, such as a Gaussian. This allows one to retain the self-interaction terms. In the case of standard vortex blobs (i.e., zeroth-order MVBs), this distinction makes no difference because the gradient of the kernel vanishes at the origin and the self-interaction term contributes nothing to the dynamics. However, the derivatives of the kernel of degree 2 and higher do not vanish at the origin. As a result, the self-interaction terms do contribute to the dynamics for MVBs of order 2 and higher. The choice to discard the self-interaction terms in Yanovsky et al. (2009), versus our choice to keep them explains one of the major discrepancies between our work and Yanovsky et al. (2009). In particular, Yanovsky et al. (2009) was concerned with generalizing the (unregularized) point vortex method in the same way that we have generalized the vortex blob method. Once the ansatz $\omega = \sum \Gamma ^{mn}_i \partial _x^m \partial _y^n \delta ( z - z_i )$ was substituted into the equations of motion, they discarded the self-interaction terms in order to handle the singularities in the velocity field. They had no other choice. Except for the initial regularization step we took, this discarding of the self-interaction term is the primary place where the derivation of the equations of motion presented here diverges from the derivation in Yanovsky et al. (2009). Discarding the self-interaction term in Yanovsky et al. (2009) leads to contradictory compatibility equations for singularities of degree 2 and higher. This is one regime where the self-interaction terms have an impact on the dynamics in our regularized formulation. One of the major findings of Yanovsky et al. (2009) was that one could avoid these contradictory compatibility conditions by limiting one’s self to combinations of point vortices and dipoles. Even in this limited scenario, our equations of motion do not match even in a regularized sense, as the vortex cores of the dipoles are not advected by the (singular) velocity field in Yanovsky et al. (2009). Additionally, as the regularization parameter goes to 0 in our framework, the velocity fields become singular, and the equations of motion for the $\Gamma$’s will explode. So we cannot expect to observe any form of convergence to the finite valued ODEs of Yanovsky et al. (2009).

## Conserved Quantities

In this section, we begin to touch upon some of the symplectic geometry of MVBs. To begin, let us consider a general vorticity distribution $\omega$. The energy is defined as

$$\begin{aligned} H(\omega ) := \frac{1}{2} \int \omega (z) G_\alpha (z-z') \omega (z') \mathrm{d}z\, \mathrm{d}z' \equiv \frac{1}{2} \int \psi (z) \omega (z) \mathrm{d}z. \end{aligned}$$

where $\psi = G_\alpha * \omega$. The vorticity equation, (1), can be seen as an instance of Hamilton’s equations on a Poisson manifold. In this case, the Poisson manifold is the space of vorticity distributions, and the Poisson bracket is the vorticity Poisson bracket derived in Marsden and Weinstein (1983). As the Hamiltonian is conserved by Hamilton’s equations, we should expect $H(\omega )$ to be constant in time. Indeed, we find that if $\omega$ satisfies (1), then

$$\begin{aligned} \frac{\mathrm{d}H}{\mathrm{d}t}(\omega )&= \frac{d}{\mathrm{d}t} \left( \frac{1}{2} \int \omega (z) G_\alpha (z-z') \omega (z') \mathrm{d}z\, \mathrm{d}z' \right) . \\&= \int (G_{\alpha } * \omega (z))\, \partial _t \omega (z) \mathrm{d}z \\&= \int \psi (z)\, \partial _t \omega (z) \mathrm{d}z \\&= \int \psi \left( \partial _x \omega \, \partial _y \psi - \partial _y \omega \, \partial _x \psi \right) \mathrm{d}z \\&= \int \partial _y \left( \frac{1}{2} \psi ^2 \right) \partial _x \omega - \partial _x \left( \frac{1}{2} \psi ^2 \right) \partial _y \omega \mathrm{d}z \end{aligned}$$

By integration by parts, we can remove the partial derivates from the $\omega$’s to find

$$\begin{aligned} = \int -\partial _{xy} \left( \frac{1}{2} \psi ^2 \right) \omega + \partial _{yx} \left( \frac{1}{2} \psi ^2 \right) \omega \mathrm{d}z = 0 \end{aligned}$$

which vanished by the equivalence of mixed partials.

As (1) is a Hamiltonian system, we can consider searching for symmetries to find other conserved quantities using Noether’s theorem. We’ve relegated the discussion of the relevant symplectic structure to Appendix 2, where derivations and proofs of the following can be found. Here we can summarize the appendix.

It’s simple to observe that the Hamiltonian *H* is translation invariant, and that *H* is rotationally invariant as long as the kernel $G_{\alpha }$ has rotational symmetry. Thus, we should expect there to be conserved quantities tied to these symmetries. We find that the quantities

$$\begin{aligned} \mathbf{J}_{\mathrm{lin}}(\omega )&= \left( \int -y \, \omega (z) \mathrm{d}z , \int x \, \omega (z) \mathrm{d}z \right) \\ \mathbf{J}_{\mathrm{ang}}( \omega )&= \int (x^2 + y^2) \omega (z) \mathrm{d}z \end{aligned}$$

are conserved. The relationship between these quantities and the symmetries of the system is explained in Appendix 2. Alternatively, one can observe the conservation of these quantities by direct calculation in the same way that conservation of energy was verified.

As the MVB ansatz is consistent with (1), we can substitute the MVB ansatz into the above conserved quantities, to obtain conserved quantities for the MVB evolution, (11) and (12). We obtain the following conserved quantities:

$$\begin{aligned} \mathbf{J}_{\mathrm{ang}}&= \sum _i \frac{\Gamma ^{0,0}_i}{2} (x_i^2 + y_i^2) - \Gamma _i^{1,0} x_i - \Gamma _i^{0,1} y_i + \Gamma _i^{2,0} + \Gamma _i^{0,2}, \\ \mathbf{J}_{\mathrm{lin}}&= \sum _i ( \Gamma _i^{0,1} - \Gamma ^{0,0}_i y_i , \Gamma ^{0,0}_i x_i -\Gamma ^{1,0}_i ), \\ H&= \sum _{m,n,\ell ,k,i} (-1)^{m+n+\ell +k} \Gamma ^{mn}_i \Gamma _j^{\ell k} \partial _{m+\ell }^x \partial _{n+k}^y G(z_i-z_j). \end{aligned}$$

Again, the first two quantities, $\mathbf{J}_{\mathrm{ang}}$ and $\mathbf{J}_{\mathrm{lin}}$, are momenta derived from Noether’s theorem for the rotational and translational symmetries of the fluid. The quantity *H* is the kinetic energy of the fluid. In Sect. 9, we will characterize the MVB dynamics as Hamiltonian systems, with Hamiltonian *H*.

To each individual MVB, there are numerous conserved quantities which can be seen as a manifestation of the conservation of circulation. To show this, let $\mathbf {u} = (u,v) = (\partial _y \psi , - \partial _x \psi )$ be a time-dependent vector field which satisfies (1). The flow of $\mathbf {u}$ is the diffeomorphism, $\Phi _t: {\mathbb {R}}^2 \rightarrow {\mathbb {R}}^2$, which sends particle labels at time 0 to their positions at time *t*. If $\omega _t$ is the vorticity at time *t*, then $\omega _t( \Phi _t(z) ) = \omega _0$ is constant in time. This conservation law can be seen as a corollary of Kelvin’s circulation theorem (Arnold and Khesin 1992). As a consequence, the quantity

$$\begin{aligned} J(t) := \int \omega _t( \Phi _t(z) ) f(z) \mathrm{d}z \end{aligned}$$

is constant in time for any $f \in C_0^\infty ({\mathbb {R}}^2)$. By applying the change of variables formula and invoking the incompressibility condition, $\det (D\Phi ) = 1$, we find

$$\begin{aligned} J(t) = \int \omega _t( z) f(\Phi _t^{-1}(z)) \mathrm{d}z. \end{aligned}$$

This form of writing *J*(*t*) makes sense when $\omega _t$ is a distribution. As a result, we find that for a vorticity of the form (8), the quantity

13 $$\begin{aligned} J(t) = \sum _{ \begin{array}{c} i \in S \\ m+n \le N \end{array} } \Gamma _i^{mn} (-1)^{m+n} \partial _x^m\partial _y^n( f \circ \Phi _t^{-1}) |_{z = z_i(t)} \end{aligned}$$

is conserved for any $f \in C_0^\infty ({\mathbb {R}}^2)$. While this conservation law holds for all functions with compact support, *f*, we do not obtain infinitely many conserved quantities when $\omega _t$ satisfies the MVB ansatz and *S* is finite. This is because the expression on the right-hand side only depends on the *N*th-order Taylor expansion of *f* at $z_i(0) \equiv \Phi _t^{-1}(z_i(t) )$, as is illustrated by the Faà di Bruno formula. We will not display the Faà di Bruno formula here because it requires nearly a page of notational definitions before to writing it down (Constantine and Savits 1996). Nonetheless, by computing the cardinality of jet spaces, one would obtain $\mathrm{card}(S) \frac{ N(N+1)}{2}$ independent conserved quantities as a result of (13). These conserved quantities can be interpreted as a finite dimensional manifestation of the conservation of circulation.

## Moments

In this section, we present how the moments of the vorticity distribution evolve in time. We will find that when the vorticity distribution is that of a MVB, and then the moments form a closed dynamical system at finite order.

The $(a,b)^{\mathrm{th}}$ moment of the vorticity, $\omega$, centered around the vortex position $(x_i,y_i) \in {\mathbb {R}}^2$ is given by

$$\begin{aligned} \mu ^{ab}_i := \int (x-x_i)^a (y-y_i)^b \omega \mathrm{d}x\mathrm{d}y. \end{aligned}$$

We call the integer $a+b$ the *order* of the moment. For a general vorticity, the evolution for the *n*th-order moments will depend on the $(n+1)$th and higher order moments and so we cannot concoct a closed dynamical system for the moments of order *n* and less. However, this is not the case if $\omega$ satisfies the MVB ansatz, and the points $(x_i,y_i)$ are given by the locations of the jet vortices. If $\omega$ satisfies the MVB ansatz (8), then

$$\begin{aligned} \mu ^{ab}_i = \sum _{ \begin{array}{c} j \in S \\ m \le a , n \le b \end{array}} (-1)^{m+n} \frac{a! b!}{(a-m)!(b-n)!} \Gamma _j^{mn} (x_j - x_i)^{a-m} (y_j - y_i)^{b-n}, \end{aligned}$$

for $a+b \le N$ and $i \in S$. Given the points $z_i \in S$, one can write the moments in terms of the circulation strengths, the $\Gamma$’s. For the moment $\mu _i^{ab}$ with $a+b \le N$ with $a,b \in \mathbb {N}$, we may invert this relationship to write $\Gamma _i^{mn} = \Gamma _i^{mn}( \mu )$, i.e., as a function of the moments. Invoking the motion equations for the $\Gamma$’s and substituting, the relation between the $\Gamma$’s and the $\mu$’s yields a closed dynamical system for the $\mu$’s.

### Remark 6.1

This relation between the $\Gamma$’s and the $\mu$’s may also be important in the context of plasma physics, especially when one recalls that (1) can be interpreted as a one-dimensional plasma model. Specifically, phase-space moments of the Vlasov probability distribution form an important dynamical link between Lagrangian-particle and Eulerian-continuum descriptions. The phase-space moments of the Vlasov probability distribution provide *collective coordinates* for the Hamiltonian dynamics of ensembles of particles. For more explanation of this property of Hamiltonian collectivization of the phase-space moments, see Guillemin and Sternberg (1990), Holm et al. (1990) and Gibbons et al. (2008a, b). In plasma dynamics, the phase-space moments arise from a Taylor expansion of the Vlasov particle distribution, taken around its centroid in phase-space. For planar incompressible flow of an ideal fluid, the phase-space comprises the (*x*, *y*) Lagrangian coordinates of a fluid particle, and the corresponding moments arise from Taylor expansions around the centroid of the (smooth) vorticity distribution. The duality between the resulting spatial moments of a smooth vorticity distribution and the MVBs corresponding to higher order singular vorticity distributions also obtained from a Taylor expansion raises the intriguing question of finding a relation between these two types of dynamical description. This question is particularly intriguing because the dynamics of moments beyond quadratic order in general does not close to form a finite dimensional Hamiltonian system, while the dynamics of MVBs closes at every order.

### Remark 6.2

There exist other systems for approximating the dynamics of moments which differ from the one presented here. In particular, the equations of motion for the moments here form a closed system at order *N*, whereas other methods for deriving dynamical systems for moments (Uminsky et al. 2012; Nagem et al. 2009; Gibbons et al. 2008a, b) require truncations in order to form a closed system. For example, Uminsky et al. (2012) approximates the stream function as a sum of Hermite functions with evolving centroids and weights. In order to obtain the evolution for the weights and the centroids, they project the viscous vorticity equation onto this space via $L^2$ projection. The resulting formulas are explicit and efficient to compute, albeit more complex than the formulas found in this paper. The primary source of error for Uminsky et al. (2012) over long times is the discrepancy between the projected evolution equations and the true evolution equations. In contrast, we approximate an Euler fluid with a regularized fluid equation which we solve exactly. This is not to say that error is not accumulated in time. The primary source of error for our method over long times is the discrepancy between the regularized fluid equations and the true fluid equations.

Admittedly, the equations of motion for the moments in Uminsky et al. (2012) bear some resemblance to the equations of motion for the $\Gamma$’s in our method. Both are quadratic in their respective variables, with coefficients involving combinatorial functions. A more precise relationship, if one exists, is difficult to discern. Philosophically, the methods share much in common. However, due to the fundamental approximation technique of projecting the equations of motion versus regularizing them, the methods are indeed distinct. This difference cascades throughout the study of both methods. For example, the convergence for Uminsky et al. (2012) is obtained via the convergence of spectral approximations, while the convergence of our method is a corollary of the convergence of a regularized fluid model (see Mumford and Michor 2013; Foias et al. 2001 for such convergence proofs).

## Numerical Aspects

In this section, we discuss various numerical aspects of using MVBs to model fluid dynamics. We will observe how MVBs can be used to reduce the number of necessary pairwise computations without a drastic compromise in accuracy. We will also present an algorithm for constructing an initial condition of MVBs from a given stream function.

### Remark 7.1

We refer to Chiu and Nicolaides (1988) for a convergence proof and error analysis of the first-order case. A convergence proof is beyond the scope of this article. Suffice it to say, such a proof would likely resemble Chiu and Nicolaides (1988).

### Grouping and Reduction of Pairwise Computations

Let us consider the vorticity distribution

$$\begin{aligned} \omega = \Gamma _1 \delta _{z_1} + \Gamma _2 \delta _{z_2}. \end{aligned}$$

If $z_1$ and $z_2$ are close, we can define the quantities ${\bar{z}} = (z_1+z_2)/2$ and $\delta z = z_1 - z_2$ to obtain the approximation

$$\begin{aligned} \int \omega (z) f(z)\mathrm{d}z&= \Gamma _1 f(z_1) + \Gamma _2 f(z_2) \\&= \Gamma _1 \left( f({\bar{z}}) + \partial _x f({\bar{z}}) \cdot \frac{\delta x}{2} + \partial _y f({\bar{z}}) \cdot \frac{\delta y}{2} \right) \\&\quad + \Gamma _2 \left( f({\bar{z}}) - \partial _x f({\bar{z}}) \cdot \frac{\delta x}{2} - \partial _y f({\bar{z}}) \cdot \frac{\delta y}{2} \right) + o( h ) \end{aligned}$$

where $h = \Vert \delta z \Vert$. Therefore, the distribution

$$\begin{aligned} {\tilde{\omega }} = \Gamma \delta _{{\bar{z}}} + \Gamma ^x \partial _x \delta _{{\bar{z}}} + \Gamma ^y \partial _y \delta _{{\bar{z}}} \end{aligned}$$

with

$$\begin{aligned} \Gamma = \Gamma _1 + \Gamma _2 \quad ,\quad \Gamma ^x = \frac{\delta x}{2} (\Gamma _2 -\Gamma _1) \quad ,\quad \Gamma ^y = \frac{\delta y}{2} (\Gamma _2 -\Gamma _1) \end{aligned}$$

serves as a *o*(*h*) approximation of $\omega$ in the sense of distributions. Moreover, the stream function ${\tilde{\psi }} := G_\delta * {\tilde{\omega }}$ is an *o*(*h*) approximation of $\psi := G_\delta * \omega$ in the traditional sense of analysis on functions.

We have just described the first case of grouping two *N*th-order MVBs concentrated at $z_1$ and $z_2$ into a single $(N+1)$th-order MVB concentrated at the average position ${\bar{z}}$. More generally, we can consider the ansatz

$$\begin{aligned} \omega = \sum _{m+n \le N} \Gamma _1^{mn} \partial _x^m \partial _y^n \delta _{z_1} + \Gamma _2^{mn} \partial _x^m \partial _y^n \delta _{z_2} \end{aligned}$$

and observe

$$\begin{aligned}&\int \omega (z) f(z)\mathrm{d}z\\&\quad = \sum _{m+n \le N} (-1)^{m+n} \left( \Gamma _1^{mn} \partial _x^m \partial _y^n f(z_1) + \Gamma _2^{mn} \partial _x^m \partial _y^n f(z_2) \right) \\&\quad = \Bigg \{ \sum _{m+n \le N} (-1)^{m+n} \Gamma _1^{mn} \left( \partial _x^m \partial _y^n f({\bar{z}}) {+} \partial _x^{m+1} \partial _y^n f(\bar{(}z)) \cdot \frac{\delta x}{2} {+} \partial _x^m \partial _y^{n+1} f(\bar{(}z)) \cdot \frac{\delta y}{2} \right) \\&\qquad + (-1)^{m+n} \Gamma _2^{mn} \left( \partial _x^m \partial _y^n f({\bar{z}}) - \partial _x^{m+1} \partial _y^n f(\bar{(}z)) \cdot \frac{\delta x}{2} - \partial _x^m \partial _y^{n+1} f(\bar{(}z)) \cdot \frac{\delta y}{2} \right) \Bigg \} \\&\quad + o( h ) . \end{aligned}$$

The above computation implies that the quantity

$$\begin{aligned}&{\tilde{\omega }} :=\sum _{m+n \le N+1} \\&\left( \Gamma _1^{mn} + \Gamma _2^{mn} - \frac{\delta x}{2}( \Gamma _1^{ m-1,n} - \Gamma _2^{m-1,n} ) - \frac{\delta y}{2} ( \Gamma _1^{m,n-1} - \Gamma _2^{m,n-1}) \right) \partial _x^m \partial _y^n \delta _{{\bar{z}}} \end{aligned}$$

serves as an *o*(*h*) approximation of $\omega$. Of course, this again implies that the corresponding stream functions are approximated to order *h* as well. Note that ${\tilde{\omega }}$ is concentrated above a single point, ${\bar{z}}$, while $\omega$ is concentrated above two points.

#### Remark 7.2

Such reductions are even more dramatic when considering higher order jets. In particular, $2^N$ zeroth-order MVBs can be approximated with a single *N*th-order MVB by applying the above approximations iteratively.

The computation of pairwise interactions in the vortex method was once a major bottleneck in implementing the standard vortex method for real-world applications. It was not until the invention of the fast multipole method that it became tractable to compute millions of pairwise interactions by reducing the complexity from an ${\mathcal {O}}(n^2)$ calculation to an ${\mathcal {O}}(n \log (n))$ calculation, where *n* is the number of vortices (Greengard and Rokhlin 1987). However, in the case of viscous fluids with boundaries, vorticity is shed from the boundaries. As a result, the vortex blob method of Chorin (1973) created new vortices at the boundary by using the Kutta condition as a creation criteria. For these applications, *n* will grow in time without bound, and some means of discarding vortices must be invoked. It is here that the grouping of MVBs could be useful. If one merges two *N*th-order MVBs to obtained a $(N+1)$th-order MVB, the amount of scalars and data typically increases. So one must still make a tough decision as to what data to discard (e.g., through some tolerance or by simply truncating at level *M*). Nonetheless, the analysis presented here could shed light on how best to implement this approach.

#### Remark 7.3

The merging of blobs of vorticity has been studied analytically (Melander et al. 1988) and numerically (Weiss and McWilliams 1993; Melander et al. 1988; Le Dizès and Verga 2002), as well as in the laboratory (Fine et al. 1991). All of this study has been in the slightly viscous (or nearly inviscid) regime. The grouping approach discussed here can be used to numerically resolve such collision events. In theory, there is no issue with collisions because we are considering regularized vortices where the induced velocity field from a single MVB is always finite. However, as $\delta$ becomes smaller, the velocity near the vortex core diverges. This should be of concern as the convergence analysis of the vortex method pre-supposes that $\delta \ll 1$. Typically such a near collision is handled by using a smaller time step (as the ODE is quite stiff). Grouping of MVBs suggests an alternative by avoiding this pairwise interaction altogether. Perhaps such an approach could be viewed as a variation of the punctuated dissipation events described in Weiss and McWilliams (1993) where an initial vorticity distribution is found to asymptotically approach a smoother axisymmetric vortex blob, and discrete vortex mergers are implemented to model this behavior.

#### Remark 7.4

There are qualitative questions which arise from mergers. For example, when two zeroth-order vortex blobs are near each other, they will typically scatter after some finite time. Merging these blobs into a single first-order blob will prohibit this scattering event from ever occurring. That both the zeroth-order MVB solution and the merged first-order MVB represent exact solutions of the fluid (after the merger event) are attributable to the long-term sensitivity to initial conditions near collision events. The scattering angle can be virtually anything since zeroth-order MVBs can waltz around each other many times before scattering. The amount of time two zeroth-order MVBs can spend waltzing around each other, and perhaps the merged solution represent some sort of limiting solution. That is to say, the merged solutions can be interpreted as the “waltzing for eternity” solution.

The irreversibility of merging is disturbing when one takes it to its extreme, one massive high order MVB. In order to address this, a means of splitting high order MVBs into lower order ones should be considered. The primary difficulty here is in determining when to split. In the case of mergers, we can decide to merge MVBs when they are close. Such a criterion is not immediately apparent in the case of splitting MVBs.

#### A Numerical Experiment with Grouping

For illustrative purposes, we can numerically group four zeroth-order MVBs into two first-order MVBs, and then one second-order MVB. In particular, we can consider the initial condition

14 $$\begin{aligned} {\left\{ \begin{array}{ll} z_0 = (-0.25,-0.25),\quad \Gamma _0 = \phantom {-}0.3 \\ z_1 = (-0.25,\phantom {-}0.25),\quad \Gamma _1 = -0.35 \\ z_2 = (\phantom {-}0.25,\phantom {-}0.25),\quad \Gamma _2 = -0.2 \\ z_3 = (\phantom {-}0.25,-0.25),\quad \Gamma _3 = \phantom {-}0.4 \\ \end{array}\right. } \end{aligned}$$

**Fig. 4:**
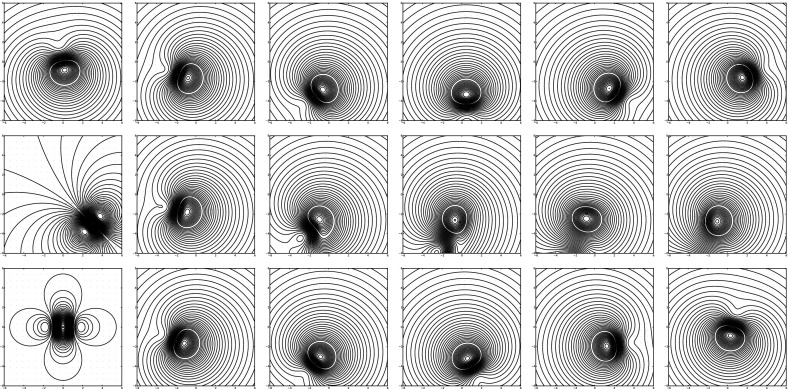
Snapshots of the evolution for various initial conditions at time $t=0,51,101,152,202,253$. The *top row* depicts the evolution of four zeroth-order MVBs given by the initial condition (14). The *middle row* depicts the evolution of two first-order MVBs obtained by grouping. The *bottom row* depicts the evolution of one second-order MVB obtained by grouping.

The corresponding dynamics are depicted in the top row in Fig. 4.

Next we group $z_1$ with $z_0$ and $z_2$ with $z_3$ in order to obtain two first-order MVBs with initial condition

15 $$\begin{aligned} {\left\{ \begin{array}{ll} z_0 = (-0.25,0.0),\quad \Gamma _0 = -0.05,\quad \Gamma ^x = 0.0, \quad \Gamma ^y = 0.1625\\ z_1 = ({-}0.25,0.0),\quad \Gamma _1 = {-}0.20, \quad \Gamma ^x = 0.0,\quad \Gamma ^y = 0.1500\\ \end{array}\right. } \end{aligned}$$

The corresponding dynamics are depicted in the middle row in Fig. 4. The dynamics appear qualitatively similar at the beginning of the evolution. Then the dynamics diverge around time $t=150$ when the two first-order MVBs separate from one another, in contrast to the dynamics of the zeroth-order MVBs.

Finally, we group the two first-order MVBs to obtain a single second-order MVB. Again, the dynamics appear qualitatively similar at the beginning of the evolution. Oddly, the dynamics of the second-order MVB appear qualitatively similar to the zeroth-order case even at $t=253$. As there is only a single vortex, the separation of vortices mentioned in the first-order MVB experiment is not possible here. As a result, the dynamics of the original zeroth-order MVB dynamics appear to be approximately recovered.

### Approximation of Initial Conditions

In this section, we will illustrate how initialize MVBs when given a stream function $\psi$ at time 0. We can begin by defining an inner product on the space of distributions on ${\mathbb {R}}^2$, given by

$$\begin{aligned} \langle \omega _1 , \omega _2 \rangle _{G_\delta } := \int \omega _1(z) G_\delta (z - {\tilde{z}}) \omega _2({\tilde{z}}) \mathrm{d}z \mathrm{d}{\tilde{z}}. \end{aligned}$$

Consequently, the energy of the fluid is given by $H(\omega ) = \frac{1}{2} \Vert \omega \Vert ^2_{G_\delta } = \frac{1}{2} \langle \omega ,\omega \rangle _{G_\delta }$.

Let *K* be a compact set, and let $0 < h \ll 1$ be small so that we may define the finite grid $\Lambda _{h} = \{ (ah,bh) \in K \mid (a,b) \in \mathbb {Z}^2 \}$.^1^ Given an $\omega \in {\mathcal {D}}'({\mathbb {R}}^2)$, we can attempt to approximate $\omega$ via Dirac deltas supported on $h \mathbb {Z}^2$. There is a natural way to do this with respect to the inner product $\langle \cdot , \cdot \rangle _{G_\delta }$. We could define $\omega _h^{(0)} = \sum _{i \in \mathbb {Z}^2} \Gamma _i \delta _{z_i}$ by requiring the error, $\omega _h^{(0)} - \omega$, to be $\langle \cdot , \cdot \rangle _{G_\delta }$-orthogonal to $\delta _z$ for each $z \in \Lambda _{h}$. This means that $G_\delta *\omega (z) = \sum _i \Gamma _i G_\delta (z-z_i)$ for each $z \in \Lambda _{h}$. Thus, $\psi _h^{(0)} = \sum _i \Gamma _i G_\delta (z-z_i)$ can be seen as a zeroth-order approximation to $\psi = G_\delta *\omega$ because $\psi _h^{(0)}(z) = \psi (z)$ for all $z \in \Lambda _{h}$. Therefore, for smooth $\omega$’s, we obtain an error of order ${\mathcal {O}}( \Delta x)$ for a grid spacing of $\Delta x$ using zeroth-order MVBs.

The same reasoning applies if we consider $\omega ^{(k)}_h = \sum _{i,m+n \le N} \Gamma _i^{mn} \partial _x^m \partial _y^n \delta _{z_i}$. We define the scalars $\Gamma _i^{mn}$ via the equations

$$\begin{aligned} \partial _x^\ell \partial _y^k \psi (z_i) = \sum _j (-1)^{m+n} \Gamma _j^{mn} \partial _x^{m+\ell } \partial _y^{n+k} G_\delta (z_i - z_j) \end{aligned}$$

for $\psi = G_\delta *\omega$, $z_i \in \Lambda _{h}$, and $|\beta | \le k$. Then $\psi ^{(k)}_h (z)= \sum _{i,\alpha } (-1)^{m+n} \Gamma _k^{mn} \partial _x^m \partial _y^n G_\delta ( z - z_i)$ serves as an order *k* approximation of $\psi$ when $\psi \in C^k$. In particular, for smooth $\omega$’s, we obtain an error of order ${\mathcal {O}}( \Delta x^{k+1})$ for a grid spacing of $\Delta x$ using *k*th-order MVBs.

As an example, we numerically compute the corresponding approximations of the stream function

16 $$\begin{aligned} \psi (x,y) = \exp ( - r^2 ) - \exp ( - r^2 / 2 ) \end{aligned}$$

**Fig. 5:**
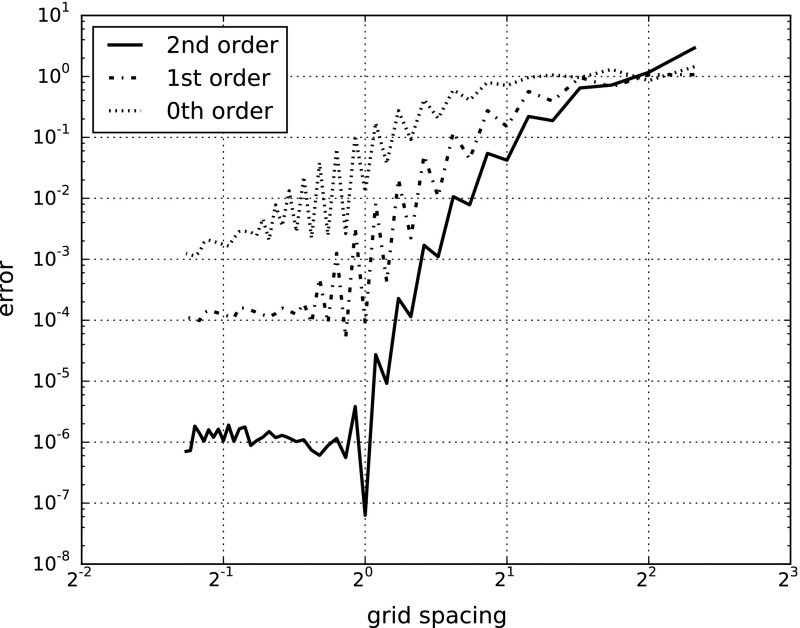
A convergence plot of the error in the sup-norm of the reconstructed stream function approximated using MVBs of order 0, 1, and 2

The results are depicted in Fig. 5 where we observe sup-norm convergence on the interior of *K*. In particular, we measure the sup-norm error on the subregion ($-3<x,y<3$) with $K = \{ (x,y) \mid -6 \le x,y \le 6 \}$. We observe convergence using MVBs at orders zero, one, and two. In each case, a grid spacing is reached where the error plateaus (possibly due to machine precision). Nonetheless, higher order MVBs appear to out perform lower order ones for smaller grid spacings. In particular, we observe slopes in a log–log plot of magnitudes 1,2, and 3, suggesting that first-, second-, and third-order convergence rates for zeroth-, first-, and second-order MVBs, respectively.

In terms of complexity, in order to achieve a desired error bound, $$e_{tol} >0 $$, you would need to use a grid with $${\mathcal {O}}( e_{tol}^{-2/(k+1)} )$$ MVBs. While the number of MVBs drops as *k* increases, one could object that a high order MVB is much more complex than a low order one. However, the number of degrees of freedom for a *k*th-order MVB is $$2 + \sum _{j=0}^{k} (2^k / k!)$$ which monotonically converges to a constant (roughly 9.39) as $$k \rightarrow \infty $$. Therefore, the number of degrees of freedom is dominated by $${\mathcal {O}}( e_{tol}^{-2/(k+1)} )$$ as well. In other words, when $$\psi $$ is highly differentiable we observe benefits in terms of complexity and storage to using a larger *k* regardless of whether one measures complexity by the number of parameters to keep track of, or the number of MVBs.

## Numerical Experiments

In these section, we present the results of numerical experiments involving small numbers of vortices, for $N=1,2$, and 3.

### Behavior of Isolated MVBs

Next, we will briefly explore the behavior of a single isolated *k*th-order MVB with $\Gamma ^{mn} = 0$ with $m+n < k$ for $k=0,1,2$. This case allows us to investigate the dynamics induced by the higher order circulation variables in the absence of the lower order ones.

### Order 0

The behavior of a single zeroth-order MVB is explicitly solvable because the dynamics are stationary.

### Order 1

**Fig. 6:**
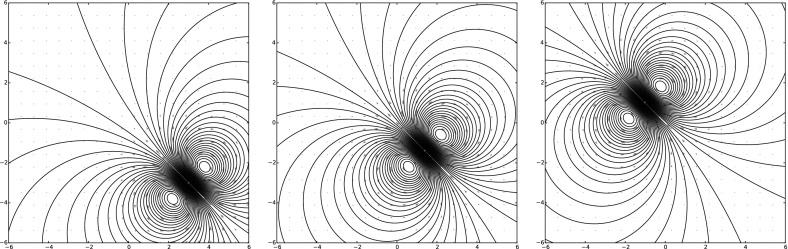
A first-order MVB with initial conditions given by (17) with snapshots taking at $t=0,10,25$

The behavior of a single first-order MVB with $\Gamma = 0$ is explicitly solvable. Given the initial condition $(x(0) , y(0) , \Gamma (0) , \Gamma ^x(0) , \Gamma ^y(0) )$ with $\Gamma (0) = 0$, we find

$$\begin{aligned}&x(t) = x(0) + v^x t,\quad \quad y(t) = y(0) + v^y t,\quad \quad \Gamma (t) = \Gamma (0) \\&\Gamma ^x(t) = \Gamma ^x(0),\quad \quad \Gamma ^y(t) = \Gamma ^y(0) \end{aligned}$$

where $v^x = \Gamma ^x(0) \partial _{xy}G_\delta (0) + \Gamma ^y(0) \partial _{yy}G_\delta (0)$ and $v_y = -\Gamma ^y(0) \partial _{xy}G_\delta (0) - \Gamma ^x(0) \partial _{xx}G_\delta (0)$. In Fig. 6, we depict such a trajectory with initial condition

17 $$\begin{aligned} x(0) = -3 , y(0) = -3 , \Gamma (0) = 0 , \Gamma ^x = 1, \Gamma ^y = 1 \end{aligned}$$

### Order 2

**Fig. 7:**
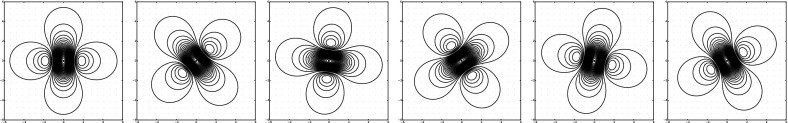
A first-order MVB with initial conditions given by (18) with snapshots taking at $t=0,5,10,15,20,25$

The behavior of a second-order vortex does not seem to be explicitly solvable. Here we consider initial conditions for which

18 $$\begin{aligned} x(0) = 0,\quad \quad y(0) = 0,\quad \quad \Gamma (0)^{xx} = 1 \end{aligned}$$

and all the other circulation variables are initially set to 0. The results are depicted in Fig. 7. We observe a structure which rigidly rotates counterclockwise.

### A Scattering Experiment

**Fig. 8:**
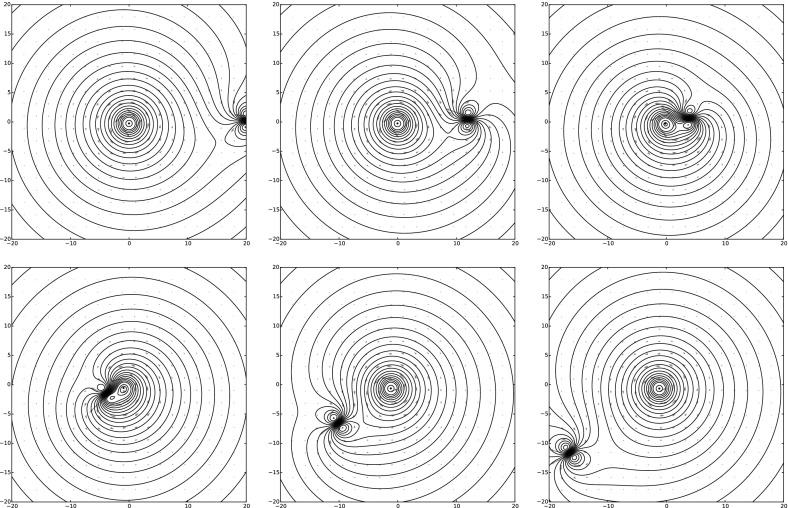
A numerical run is shown with mirror-image initial conditions for two first-order MVBs, as given in (19). From left to right and top to bottom, these are snapshots at times $t=0,25,50,75,100,125$, respectively,

Next we consider two MVBs. The first is a first-order MVB with an initial velocity pointed just slightly above origin. The second MVB is a standard zeroth-order vortex located at the origin. Specifically, we consider the initial conditions

19 $$\begin{aligned} {\left\{ \begin{array}{ll} z_0 = (20.0 , {-}0.25), \quad \Gamma _0^{0,0} = 0.0 \quad ,\quad \Gamma _0^{1,0} = 0.0,\quad \Gamma _0^{0,1} = -1.0\\ z_1 = (20.0 , -0.25), \quad \Gamma _0^{0,0} = 1.0,\quad \Gamma _0^{1,0} = 0.0,\quad \Gamma _0^{0,1} = {-}0.0 \end{array}\right. } \end{aligned}$$

with $\Gamma _i^{mn} = 0$ for $m+n > 1$ and $i=0,1$. The vortex at the origin appears to remain at the origin throughout the numerical run ($t=0$ to $t=150$). The first-order MVB starts by moving to the left in a straightline until it comes into proximity of the zeroth-order vortex. Then the first-order MVB swings around the zeroth-order vortex, traversing an angle of roughly 30 degrees before zooming off into the lower left quadrant of the plane in a straight line. These results are depicted in Fig. 8.

### The Method of Images

Here we incorporate first-order MVBs into the method of images (Jackson 1975; Smith 2011). We consider the initial conditions consisting of two first-order MVBs which are mirror images of each other with respect to the *x*-axis. By symmetry, the resulting vector field should be tangential to the *x*-axis and provides a means of considering a boundary that satisfied the no-penetration condition. Specifically, we consider the initial condition:

20 $$\begin{aligned} {\left\{ \begin{array}{ll} z_0 = (1.5 , {-}1.5), \quad \Gamma _0^{0,0} = {-}0.5,\quad \Gamma _0^{1,0} = {-}0.5,\quad \Gamma _0^{0,1} = 1.5 \\ z_1 = (1.5,-1.5), \quad \Gamma _0^{0,0} = -0.5,\quad \Gamma _0^{1,0} = -0.5,\quad \Gamma _0^{0,1} =1.5 \end{array}\right. } \end{aligned}$$

with $\Gamma _i^{mn} = 0$ for $m+n > 1$ and $i=0,1$.

**Fig. 9:**
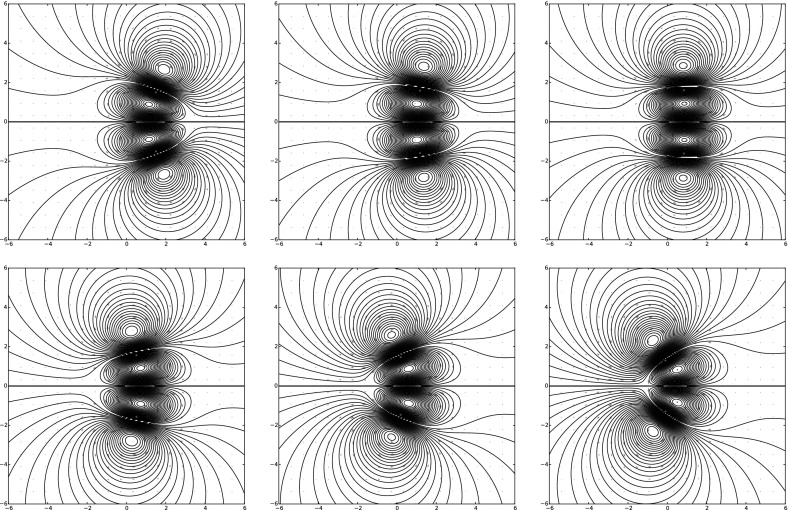
Numerical results are shown for two first-order MVBs with mirror-image initial conditions given by (20). From *left* to *right* and *top* to *bottom*, these are snapshots at times $t=0,1.7,3.4,5,6.7,8.4$, respectively. Apparently, a first-order MVB reflects elastically from a fixed boundary, so that its angle of reflection equals its angle of incidence

The resulting dynamics depicted in Fig. 9 shows that as a first-order MVB approaches a boundary, it will turn its motion along the boundary and then move away so that its angle of reflection equals its angle of incidence.

## Hamiltonians and Symplectic Structures

In modern Hamiltonian mechanics, as described in Abraham and Marsden (1978) and Arnold (2000), the Hamiltonian is a function on a symplectic manifold, which produces equations of motion. An important instance of a symplectic manifold is a coadjoint orbit (defined below). In this section, we compute the coadjoint orbit of a MVB as well as the associated symplectic structure. The coadjoint orbit of an initial vorticity distribution $\omega _0$ comprises the set

$$\begin{aligned} {{\mathrm{Orb}}}(\omega _0) := \{ \omega _0 \circ \varphi ^{-1} \mid \varphi \in {{\mathrm{SDiff}}}({\mathbb {R}}^2) \}. \end{aligned}$$

In fact, ${{\mathrm{Orb}}}(\omega _0)$ inherits the structure of a smooth manifold, and a tangent vector on ${{\mathrm{Orb}}}(\omega _0)$ at the point ${\tilde{\omega }} \in {{\mathrm{Orb}}}(\omega )$ is given by a distribution of the form $\pounds _{\mathbf {u}}[{\tilde{\omega }}] := u \partial _x {\tilde{\omega }} + v \partial _y {\tilde{\omega }}$ for some (nonunique) divergence-free vector field $\mathbf {u} = (u,v) \in {\mathfrak {X}}_{\mathrm{div}}({\mathbb {R}}^2)$. The symplectic structure is nothing more than a special case of the one derived via the Kirillov–Kostant–Souriau theorem (Abraham and Marsden 1978, see the boxed formula on p.303). In particular, the symplectic structure on ${{\mathrm{Orb}}}(\omega )$ is given by

21 $$\begin{aligned} \Omega _\omega ( \pounds _{\mathbf {u}_1}[\omega ] , \pounds _{\mathbf {u}_2}[\omega ] ) = \int \omega (z) ( u_1(z) v_2(z) - v_1(z) u_2(z) ) \mathrm{d}z. \end{aligned}$$

When $\omega$ is a smooth distribution, the symplectic structure may be identified with a differential 2-form and this formula matches the symplectic form derived on page 313 of Marsden and Weinstein (1983). In the case that $\omega$ satisfies the ansatz (8), we find that given any $\varphi \in {{\mathrm{SDiff}}}({\mathbb {R}}^2)$ that

$$\begin{aligned} \int \omega _0(\varphi ^{-1}(z)) f(z) \mathrm{d}z&= \int \omega _0(z) , f (\varphi (z)) \mathrm{d}z \\&= \gamma _i^\alpha \partial _{\alpha }|_{z=Z_i} (f \circ \varphi )(z). \end{aligned}$$

Here we have used the change of variables formula and the fact that $\det ( D\varphi ) = 1$. By the multivariate Faá di Bruno formula, the expression $\partial _{\alpha }|_{z=Z_i} (f \circ \varphi )(z)$ is a sum of the partial derivatives of *f* at the points $\varphi (Z_i)$ of order less than that of the multi-index $\alpha$ (Constantine and Savits 1996). Thus, $\omega _0 \circ \varphi ^{-1}$ is contained in the finitely parametrized subset $M^{(k)} := \{ \sum _{|\alpha | \le k} \Gamma _i^\alpha \partial _{\alpha } \delta _{Z_i} \}$ for any $\varphi \in {{\mathrm{SDiff}}}( {\mathbb {R}}^2)$. Therefore, ${{\mathrm{Orb}}}(\omega _0)$ is a finite dimensional manifold when $\omega _0$ satisfies the jet vortex ansatz.

Having identified a symplectic manifold, ${{\mathrm{Orb}}}(\omega _0)$, we can then ask the question *“are the dynamics Hamiltonian on* ${{\mathrm{Orb}}}(\omega _0)$?” Of course, the answer is *“yes”*. This is the primary content of Marsden and Weinstein (1983). We provide our own explanation here for convenience.

For a general vorticity distribution $\omega$, we may consider the kinetic energy Hamiltonian

22 $$\begin{aligned} H(\omega ) = \frac{1}{2} \int \omega (z) G_\delta (z-{\tilde{z}}) \omega ({\tilde{z}}) \mathrm{d}z d{\tilde{z}}. \end{aligned}$$

where $\omega$ may be of the form (8). In order to find Hamilton’s equations on ${{\mathrm{Orb}}}(\omega _0)$ choose some $\omega \in {{\mathrm{Orb}}}(\omega _0)$ and calculate the vector $X_H(\omega )$ tangent to ${{\mathrm{Orb}}}(\omega _0)$ given by Hamilton’s equations. It must be the case that $X_H(\omega ) = \pounds _{\mathbf {u}}[\omega ]$ for some (nonunique) vector field $\mathbf {u} = (u,v) \in {\mathfrak {X}}_{\mathrm{div}}({\mathbb {R}}^2)$. Our goal is to solve for $\mathbf {u}$. By the definition of the Hamiltonian vector field $X_H$, we see that for any $\mathbf {u}' = (u',v') \in {\mathfrak {X}}_{\mathrm{div}}({\mathbb {R}}^2)$

$$\begin{aligned} \int \omega (z) \left( u(z) v'(z) - v(z) u'(z) \right) \mathrm{d}z&= \Omega _{\omega }( \pounds _{\mathbf {u}}[\omega ] , \pounds _{\mathbf {u}'}[\omega ] ) {=}{-}\int \frac{\delta H}{\delta \omega } (z) \left( \pounds _{\mathbf {u}'}[\omega ] \right) (z) \mathrm{d}z \\&= - \int G_\delta (z-{\tilde{z}}) \omega ({\tilde{z}}) \left( \pounds _{\mathbf {u}'}[\omega ] \right) (z) \mathrm{d}{\tilde{z}} \mathrm{d}z . \end{aligned}$$

If we let $\psi := G_\delta *\omega = \int G_\delta (\cdot -{\tilde{z}}) \omega ({\tilde{z}}) \mathrm{d} {\tilde{z}}$, then integration by parts implies

$$\begin{aligned} \int \omega (z) \pounds _{\mathbf {u}'}[\psi ](z) = \int \omega (z) \left( u'(z) \partial _x \psi (z)+ v'(z) \partial _y \psi (z) \right) \mathrm{d}z. \end{aligned}$$

We see that $\mathbf {u} = (-\partial _y \psi ,\partial _x \psi )$ is one possible solution. As $\Omega$ is nondegenerate on the tangent spaces of ${{\mathrm{Orb}}}(\omega )$, this is the unique solution. As a result, the evolution prescribed by $X_H$ is precisely (1). This proves that (1) can be seen as a Hamiltonian equation on ${{\mathrm{Orb}}}(\omega _0)$ with respect to the symplectic structure (21) and the Hamiltonian (22).

### The First-Order Case

Let us illustrate these Hamiltonian results for the case of the first-order MVB. Let $z_1,\ldots ,z_n \in {\mathbb {R}}^2$ be distinct and define the initial vorticity distribution

$$\begin{aligned} \omega _0 = \sum _{i=1}^N \gamma _i \delta _{z_i} + \gamma _i^x \partial _x \delta _{z_i} + \gamma _i^y \partial _{y} \delta _{z_i}. \end{aligned}$$

We desire to determine the coadjoint orbit, ${{\mathrm{Orb}}}(\omega _0)$, and the symplectic structure.

Indeed, we find that for any function *f*

$$\begin{aligned} \int \omega \left( \varphi ^{-1}(z)\right) f(z)\mathrm{d}z&:= \int \omega (z)f\left( \varphi (z)\right) \mathrm{d}z \\&= \gamma _i f(\varphi (Z_i)) \\&\quad - \gamma _i^x \partial _x \varphi ^x|_{z=Z_i} \partial _x f |_{z=\varphi (Z_i)} - \gamma _i^x \partial _x \varphi ^y|_{z=Z_i} \partial _y f |_{z=\varphi (Z_i)} \\&\quad - \gamma _i^y \partial _y \varphi ^x|_{z=Z_i} \partial _x f |_{z=\varphi (Z_i)} - \gamma _i^y \partial _y \varphi ^y|_{z=Z_i} \partial _y f |_{z=\varphi (Z_i)} \end{aligned}$$

Collecting like terms we find

$$\begin{aligned} \omega \circ \varphi ^{-1} = \gamma _i \delta _{\varphi (Z_i)} + \Gamma _i^x \partial _x \delta _{\varphi (Z_i)} + \Gamma _i^y \partial _y \delta _{\varphi (Z_i)} \end{aligned}$$

where

$$\begin{aligned} \Gamma = \begin{bmatrix} \Gamma _i^x \\ \Gamma _i^y \end{bmatrix} = D\varphi (Z_i) \cdot \begin{bmatrix} \gamma _i^x \\ \gamma _i^y \end{bmatrix} \end{aligned}$$

By varying $\varphi$, we can obtain any collection of distinct points $z_1,\ldots ,z_n \in {\mathbb {R}}^2$ and any collection of nonzero vectors $\Gamma _1,\ldots ,\Gamma _n \in {\mathbb {R}}^2 \backslash \{0\}$. This proves

$$\begin{aligned} {{\mathrm{Orb}}}(\omega _0)&= \left\{ \sum _{i=1}^n \gamma _i \delta _{z_i} + \Gamma _i^x \partial _x \delta _{{\tilde{z}}_i} + \Gamma _i^y \partial _{y} \delta _{z_i} \mid z_i \in {\mathbb {R}}^2, (\Gamma _i^x,\Gamma _i^y) \in {\mathbb {R}}^2 \backslash \{0\} \right\} \\&\cong \{ (z_1,\ldots ,z_n,\Gamma _1,\ldots ,\Gamma _n) \mid z_i \in {\mathbb {R}}^2, \Gamma _i \in {\mathbb {R}}^2 \backslash \{0\} , ( i \ne j \implies z_i \ne z_j ) \}. \end{aligned}$$

To derive the symplectic structure, recall the symplectic structure for a general vorticity (21). Now let $\omega = \gamma _i \delta _{z_i} + \Gamma _i^x \partial _x\delta _{z_i} + \Gamma _i^y \partial _y \delta _{z_i}$. In this case, the left-hand side of (21) can be computed with respect to divergence-free vector field $\mathbf {u} = (u,v)$ and $\mathbf {u}' = (u',v')$ as

$$\begin{aligned} \int \omega (z) \left( u v' - v u' \right) (z) \mathrm{d}z&= \gamma _i ( u(z_i)v'(z_i) - v(z_i) u'(z_i) ) \\&\quad + \Gamma _i^x ( u_{,x} v' + u {v'}_{,x} - {u'}_{,x}v - u' v_{,x})|_{z = z_i} \\&\quad + \Gamma _i^y ( u_{,y} v' + u {v'}_{,y} - {u'}_{,y}v - u' v_{,y})|_{z = z_i} \end{aligned}$$

Note that this is written entirely in terms of the first-order Taylor expansion of $\mathbf {u}$ and $\mathbf {u}'$ evaluated at $z_i$. Moreover, $\pounds _{\mathbf {u}}[\omega ] = \gamma _0 u(z_i) \partial _x \delta _{z_i} + \dots$ also has the property that it only depends on the first-order Taylor expansion of *u* and *v* at the points $z_1,\dots ,z_n$. Therefore, both sides of (21) can be written as a function of the finite collection of numbers $u(z_i), Du(z_i), v(z_i),Dv(z_i)$. The result then follows by identifying the scalars

$$\begin{aligned} u(z_i) \mapsto u_{z_i}\\ u^x_{,x}(z_i) \Gamma ^x_i + u^x_{,y}(z_i) \Gamma ^y_i \mapsto {\dot{\Gamma }}_i^x \\ u^y_{,x}(z_i) \Gamma ^x_i + u^y_{,y}(z_i) \Gamma ^y_i \mapsto {\dot{\Gamma }}_i^y. \end{aligned}$$

This proves that the symplectic structure on ${{\mathrm{Orb}}}(\omega _0)$ is more concretely written as

23 $$\begin{aligned} \begin{aligned} \Omega ( ({\dot{z}},{\dot{\Gamma }}), (\delta z,\delta \Gamma ) )&= \gamma _i ( {\dot{x}}_i \cdot \delta y_i - \delta x_i \cdot {\dot{y}}_i ) \\&\quad + {\dot{\Gamma }}_i^x \cdot \delta y_i - {\dot{\Gamma }}_i^y \cdot \delta x_i + \delta \Gamma _i^y \cdot {\dot{x}}_i - \delta \Gamma _i^x \cdot {\dot{y}}_i \end{aligned} \end{aligned}$$

In essence, we have determined a finite dimensional Hamiltonian system whose solutions solve (1) when $\psi$ is related to $\omega$ via an appropriate regularization.

#### Remark 9.1

The use of this symplectic structure shows that the map $(z_i, \Gamma _i,\Gamma _i^x,\Gamma _i^y) \mapsto \omega \in {{\mathrm{Orb}}}(\omega )$ is a symplectic momentum map.

#### Remark 9.2

The corresponding Poisson bracket can be represented in tabular form by:

**Table:** 

$\{ \cdot , \cdot \}$	*x*	*y*	$\Gamma$	$\Gamma ^x$	$\Gamma ^y$
*x*	0	1	0	0	1
*y*	−1	0	0	−1	0
$\Gamma$	0	0	0	0	0
$\Gamma ^x$	0	−1	0	0	1
$\Gamma ^y$	1	0	0	−1	0

The way to use this table is as follows. Let $H = H(\xi )$ be our Hamiltonian where $\xi = (x_1,\dots ,x_n,y_1,\ldots ,y_n,\Gamma _1,\ldots ,\Gamma _n,\Gamma _1^x,\ldots ,\Gamma _n^x,\Gamma _1^y,\dots ,\Gamma _n^y)$. Hamilton’s equations are then given by

$$\begin{aligned} \frac{\mathrm{d}f}{\mathrm{d}t} = \sum _{i,j} B^{ij} \frac{ \partial f}{\partial \xi ^i} \frac{ \partial H}{\partial \xi ^j}, \end{aligned}$$

for any function *f*, where $B^{ij}$ denotes the corresponding entry of the table. In particular, when $f=\xi ^i$, one recovers the equations of motion for the dynamics of the positions and strengths for a set of *n* first-order MVBs. Poisson geometers call $B^{ij}$ a *Poisson tensor* Abraham and Marsden (1978).

## Conclusion

In this paper, we have considered a generalization of the standard vortex blob method, obtained by augmenting the vortices with higher order circulation variables and dubbing them *multipole vortex blobs* (MVBs). By viewing the vorticity equation as an advection equation, we have obtained equations of motion for these MVBs.

The extra degrees of freedom of MVBs resulted in richer dynamics near the vortex core. Moreover, these new vorticity carrying elements exhibited a variety of novel types of solution behavior. We also observed faster convergence rates in space using higher order MVBs. Moreover, we proposed a scheme to decrease the number of pairwise interactions, by grouping MVBs of lower order into a smaller number of MVBs of higher order. Lastly, the implications of Kelvin’s circulation theorem were substantially richer in the case of MVBs than they were for the standard vortex blob method.

We have demonstrated the behavior of the MVBs with a sequence of simple numerical experiments consisting of small numbers of MVBs of various degrees. We found that first-order MVBs correspond to sums of vortex blobs and regularized dipoles which simply propagate themselves forward, while the second-order circulation variables activate richer (non-propagating) dynamics near the vortex core.

Finally, we derived the symplectic structure of MVBs using methods from Marsden and Weinstein (1983). The resulting structure turned out to be a direct generalization of the standard symplectic structure for vortex blobs.

The multiscale nature of ideal fluids is the principal obstacle to obtaining accurate models (Chorin 1994, Chapter 3). The use of MVBs augments the standard vortex blob method by allowing for singular vorticity distributions which model dynamics below the regularization length scale (i.e., at order $\delta ^k$ with $\delta \ll 1$ for a *k*th-order jet vortex). As the dynamics of MVBs are relatively easy to derive, and their analysis is tractable, we believe that MVBs will be of considerable value in understanding the place of regularized fluid models within the computational fluids community at large and they should provide renewed interest in the vortex blob method.

Future avenues of inquiry could include:

- MVBs on manifolds, such as the sphere
- The convergence properties of the MVB method
- How does one choose the regularization length scale in relation to the grid resolution. This relationship is addressed quite well for zeroth-order MVBs in Beale and Majda (1982). It is not clear if higher order MVBs change those results.
- An investigation of the kinetic theory of MVBs.

## Appendix 1: Distributions

The vorticity, $\omega$, should be viewed as a distribution, and the term “$\partial _x \omega$” should be viewed as a distributional derivative. When $\omega \in {\mathcal {D}}'({\mathbb {R}}^2)$ is a smooth distribution, there is little harm in naively interpreting $\omega$ as a smooth function on ${\mathbb {R}}^2$. However, when $\omega$ is not smooth (e.g., a Dirac delta distribution), then one needs to invoke the mathematics of distributions as distinct from that of real-valued functions. Therefore, we have included this appendix to remind the reader of the basic theory of distributions. The main reference for this section is Hörmander (2003).

The space of distributions ${\mathcal {D}}'({\mathbb {R}}^2)$ is the dual vector space to the space of smooth functions with compact supper $C^\infty _0({\mathbb {R}}^2)$. Therefore, a distribution is defined by how it maps functions to real numbers.

The distributional derivative of a given distribution $\omega \in {\mathcal {D}}'({\mathbb {R}}^2)$ in the *i*th coordinate direction may be defined as the distribution $\partial _i \omega$ obtained by

$$\begin{aligned} \int \partial _i \omega (z) f(z) \mathrm{d}z = - \int \omega (z) \partial _i f (z) \mathrm{d}z. \end{aligned}$$

For example, the Dirac delta distribution, $\delta _0$, is defined as the unique distribution such that

$$\begin{aligned} \int \delta _0(z) , f (z) \mathrm{d}z = f(0), \quad \forall f \in C_0^\infty ({\mathbb {R}}^2). \end{aligned}$$

The distributional derivative, $\partial _i \delta _0$, is given by

$$\begin{aligned} \int \partial _i \delta _0 (z) f(z)\mathrm{d}z = -\partial _i f(0), \quad \forall f \in C_0^\infty ({\mathbb {R}}^2). \end{aligned}$$

Given a distribution $\omega \in D({\mathbb {R}}^2)$ and a function $g \in C_0^\infty ({\mathbb {R}}^2)$, one can define the distribution $g \, \omega$ as

$$\begin{aligned} \int (g \, \omega )(z) f(z) \mathrm{d}z = \int \omega (z) g(z)f(z)\mathrm{d}z, \quad \forall f \in C_0^\infty ({\mathbb {R}}^2). \end{aligned}$$

For example, we find that $g \, \delta _0 = g(0) \delta _0$. A slightly more involved, but standard, example is given by the computation of $g\, \partial _i \delta _0$. We find

$$\begin{aligned} \int (g \, \partial _i \delta _0) (z) f(z) \mathrm{d}z = \int \partial _i \delta _0(z) g(z)f(z) \mathrm{d}z = - g(0) \partial _i f(0) - \partial _ig(0) f(0). \end{aligned}$$

Therefore,

$$\begin{aligned} g \, \partial _i \delta _0 = g(0) \partial _i \delta _0 - \partial _i g(0) \delta _0. \end{aligned}$$

On the left-hand side, note that *g*(0) and $\partial _i g(0)$ are merely real numbers, which are multiplying the distributions $\partial _i \delta _0$ and $\delta _0$. More generally, we find

$$\begin{aligned} \int (g\, \partial _x^m \partial _y^n \delta _0)(z) f(z) \mathrm{d}z&= (-1)^{m+n} \partial _x^m \partial _y^n (fg)(0) \\&= (-1)^{m+n} \sum _{\ell ,k=0}^{m,n} \left( {\begin{array}{c}m\\ \ell \end{array}}\right) \left( {\begin{array}{c}n\\ k\end{array}}\right) \left( \partial _{x}^{\ell } \partial _y^k f(0) \right) \left( \partial _{x}^{m-\ell } \partial _y^{n-k} g(0) \right) \end{aligned}$$

which means

24 $$\begin{aligned} g \, \partial _x^m \partial _y^n \delta _0 = (-1)^{m+n} \sum _{\ell ,k=0}^{m,n} (-1)^{\ell + k} \left( {\begin{array}{c}m\\ \ell \end{array}}\right) \left( {\begin{array}{c}n\\ k\end{array}}\right) \left( \partial _{x}^{m-\ell } \partial _y^{n-k} g(0) \right) \partial _{x}^{\ell } \partial _y^k \delta _0. \end{aligned}$$

## Appendix 2: Symmetries and Conservation Laws

The main reference for the material presented in this section is Abraham and Marsden (1978). Let *G* be a Lie group with Lie-algebra ${\mathfrak {g}}$. We will denote the dual of ${\mathfrak {g}}$ by ${\mathfrak {g}}^*$. A (left) group action of *G* on a manifold *S* is a map $\varrho : G \times S \rightarrow S$ such that $\varrho ( g h , x) = \varrho (g , \varrho (h , x))$ for all $g,h, \in G$ and $x \in S$.

### Remark 10.1

The group action $\varrho$ is not to be confused with the fluid density, often denoted as $\rho$ in fluid mechanics. This appendix relates to more general mathematical constructions which are useful, but not necessarily in the usual purview of fluid mechanics. In particular, the symbol $\varrho$ is the Greek letter “r,” which refers to the word “representation” as in “representation theory.”

One can also construct a group action, $D\varrho : G \times TS \rightarrow TS$, given by $D\varrho ( g , u ) := \left. \frac{d}{dt}\right| _{t=0} \varrho (g, x(t) )$ for $u = \frac{dx}{dt} \in T_x S$ and $g \in G$. There is also a natural Lie-algebra action, which one could also denote by $\varrho : {\mathfrak {g}} \times S \rightarrow TS$ defined by $\varrho ( \xi , x) := \left. \frac{d}{d \epsilon } \right| _{\epsilon =0} \varrho ( g_\epsilon , x)$. In particular, the map $\varrho ( \xi , \cdot ) : S \rightarrow TS$ is a vector field on *S* which we call the *infinitesimal generator* of $\xi$. When no confusion arises, it is typical to use the notation $g \cdot x$, $g \cdot u$, and $\xi \cdot x$ to denote $\varrho (g,x)$,$D\varrho (g,u)$, and $\varrho (\xi ,x)$, respectively. Finally, if $(S,\Omega )$ is a symplectic manifold, then we say that *G* acts *symplectically* (or *canonically*) if $\Omega _{g \cdot x}( g \cdot u , g \cdot v) = \Omega _x(u,v)$ for all $g \in G$, $u,v \in T_x S$ and $x \in S$.

Let us now recall the notion of a momentum map (Abraham and Marsden 1978, Definition 4.2.1). Given a symplectic manifold $(S,\Omega )$ and a Lie group *G* which acts on $(S,\Omega )$ symplectically, a momentum map is a map $\mathbf{J}:S \rightarrow {\mathfrak {g}}^*$ such that

$$\begin{aligned} d \langle \mathbf{J} , \xi \rangle = i_{\xi _S} \Omega , \end{aligned}$$

where $\langle \mathbf{J} , \xi \rangle$ denotes the real-valued function on *S* obtained by pairing $\mathbf{J}$ with an arbitrary $\xi \in {\mathfrak {g}}$, and where $\xi _S \in {\mathfrak {X}}(S)$ is the infinitesimal generator of $\xi$ on *S*. Equivalently, we could express the previous condition as

25 $$\begin{aligned} d \langle \mathbf{J},\xi \rangle (x) \cdot v_x = \Omega ( \xi \cdot x , v_x ) \end{aligned}$$

for all $\xi \in {\mathfrak {g}}$, $x \in S$ and $v_x \in T_xS$. Momentum maps are significant for a number of reasons. In particular, given a Hamiltonian on *S* with *G*-symmetry, the momentum map $\mathbf{J}$ will be conserved under the evolution of Hamilton’s equations (Abraham and Marsden 1978, Theorem 4.2.2). This is the Hamiltonian version of Noether’s theorem.

In our case, $S = \mathrm{Orb}(\omega _0) = \{ \omega _0 \circ \varphi ^{-1} \mid \varphi \in {{\mathrm{SDiff}}}({\mathbb {R}}^2) \}$ is a coadjoint orbit of some vorticity distribution on ${\mathbb {R}}^2$. Tangent vectors on *S* are of the form

$$\begin{aligned} \pounds _{\mathbf {u}} [\omega ] := u \partial _x \omega + v \partial _y \omega \end{aligned}$$

for a (perhaps nonunique) divergence-free vector field $\mathbf {u} = (u,v)$. Under this identification, the symplectic form at some $\omega \in S$ is given by an application of Kostant’s formula Abraham and Marsden (1978). This is derived in Sect. 9 and found to be

$$\begin{aligned} \Omega _\omega ( \pounds _{\mathbf {u}_1}[\omega ] , \pounds _{\mathbf {u}_2} [\omega ] ) := \int \omega (z) ( \mathbf {u}_1(z) \times \mathbf {u}_2(z) ) \mathrm{d}z. \end{aligned}$$

where $\mathbf {u}_1$ and $\mathbf {u}_2$ are divergence-free vector fields, and $\times$ denotes the planar cross-product. Here we interpret the planar cross-product as taking values in the space of real numbers so that $\mathbf {u}_1 \times \mathbf {u}_2$ is merely a smooth function.

We now will translate formula (25) to this more specific scenario. Assume *G* acts upon ${\mathbb {R}}^2$, then *G* also acts upon distributions and upon *S* by symplectic group actions. In this context, a momentum map $\mathbf{J}$ associated with a *G*-action is defined by the equation

26 $$\begin{aligned} \int \frac{\delta \langle \mathbf{J} , \xi \rangle }{\delta \omega } (z) \left( \pounds _{\mathbf {u}}[\omega ] \right) (z) \mathrm{d}z = \int \omega (z) \left( (\xi \cdot z) \times \mathbf {u}(z) \right) \mathrm{d}z \end{aligned}$$

where $\xi \cdot z$ denotes the action of $\xi \in {\mathfrak {g}}$ on $z \in {\mathbb {R}}^2$, and “$\times$” denotes the planar cross-product.

### Translational Symmetry and $\mathbf{J}_{\mathrm{lin}}$

The group ${\mathbb {R}}^2$ acts upon ${\mathbb {R}}^2$ by translation. That is to say, by sending any ${\tilde{z}} \in {\mathbb {R}}^2$ to $z + {\tilde{z}} \in {\mathbb {R}}^2$ for any ${\tilde{z}} \in {\mathbb {R}}^2$. This fact induces an action on smooth functions. In particular, there is a natural (right) action on $C^{\infty }({\mathbb {R}}^2)$ sending the function $\phi (z)$ to the function $({\tilde{z}})^*\phi (z) := \phi (z-{\tilde{z}})$. We denote the inverse operation by $({\tilde{z}})_* \phi (z) := \phi ( z + {\tilde{z}})$. This induces a (left) action on distributions which sends $\omega \in {\mathcal {D}}'({\mathbb {R}}^2)$ to the distribution $({\tilde{z}})_*\omega (z) := \omega ( z +{\tilde{z}})$. As a translation of ${\mathbb {R}}^2$ by ${\tilde{z}}$ is a volume-preserving diffeomorphism, we see that the coadjoint orbit $S \subset {\mathcal {D}}({\mathbb {R}}^2)$ is invariant under this action. Moreover, we observe the action, restricted to *S*, is symplectic because

$$\begin{aligned} \Omega _{ {\tilde{z}}_* \omega }( {\tilde{z}}_*\pounds _{u}[ \omega ] , {\tilde{z}}_*\pounds _v[\omega ] )&= \Omega _{{\tilde{z}}_* \omega } ( \pounds _{{\tilde{z}}_*u}[ {\tilde{z}}_*\omega ] , \pounds _{{\tilde{z}}_*v}[{\tilde{z}}_*\omega ] ) \\&= \int {\tilde{z}}_* \omega (z) \left( {\tilde{z}}_*(u \times v) \right) (z) dz \\&= \int \omega ( z+{\tilde{z}}) \left( u \times v \right) (z + {\tilde{z}}) dz \\&= \int \omega (z) \left( u \times v \right) (z) \mathrm{d}z \\&= \Omega _{\omega }( \pounds _u[\omega ], \pounds _v[\omega ]). \end{aligned}$$

Given this symplectic action, we can seek a momentum map, $\mathbf{J}_{\mathrm{lin}}:S \rightarrow ({\mathbb {R}}^2)^*$. Consider an arbitrary element of the Lie-algebra $\delta {\tilde{z}} = (\delta {\tilde{x}}, \delta {\tilde{y}}) \in {\mathbb {R}}^2$ and use (26) to obtain

$$\begin{aligned} \int \frac{\delta \langle \mathbf{J}_{\mathrm{lin}} , \delta {\tilde{z}} \rangle }{\delta \omega }(z) \pounds _{\mathbf {u}}[\omega ] \mathrm{d}z = \int \omega (z) \left( \delta {\tilde{x}} \, v(z) - \delta {\tilde{y}} \, u(z) \right) \mathrm{d}z \end{aligned}$$

We can rewrite the right-hand side as

$$\begin{aligned} = \int \omega (z) \pounds _{\mathbf {u}} [ \delta {\tilde{x}} \, y - \delta {\tilde{y}} \, x] \mathrm{d}z \end{aligned}$$

and upon integrating by parts this is equivalent to

$$\begin{aligned} = - \int \pounds _{\mathbf {u}}[\omega ](z) \left( \delta {\tilde{x}}\, y - \delta {\tilde{y}}\, x \right) \mathrm{d}z \end{aligned}$$

Therefore, “cancelling” the arbitrary vector $\pounds _v[\omega ]$ from both sides we find

$$\begin{aligned} \frac{\delta \langle \mathbf{J}_{\mathrm{lin}} , \delta {\tilde{z}} \rangle }{\delta \omega } = \delta {\tilde{y}} x - \delta {\tilde{x}} y \end{aligned}$$

Integrating by $\omega$ we find

$$\begin{aligned} \mathbf{J}_{\mathrm{lin}}(\omega ) = \left( -\int \omega (z) y \mathrm{d}z, \quad \int \omega (z) x \mathrm{d}z \right) \in ({\mathbb {R}}^2)^* \equiv {\mathbb {R}}^2 \end{aligned}$$

If $\omega$ satisfies the MVB ansatz

$$\begin{aligned} \omega = \Gamma _i \delta _{z_i} + \Gamma _i^x \partial _x \delta _{z_i} + \Gamma _i^y \partial _y \delta _{z_i} + \cdots \end{aligned}$$

then

$$\begin{aligned} \mathbf{J}_{\mathrm{lin}}(\omega ) = \sum _i ( \Gamma _i^{y} - \Gamma _i y_i , \Gamma _i x_i -\Gamma _i^{x} ). \end{aligned}$$

The terms of the MVBs beyond the first order do not influence $\mathbf{J}_{\mathrm{lin}}$.

### Rotational Symmetry and $\mathbf{J}_{\mathrm{ang}}$

The group ${{\mathrm{SO}}}(2)$ acts upon ${\mathbb {R}}^2$ by rotations about the origin sending $z \in {\mathbb {R}}^2$ to $R_\theta \cdot z := ( \cos (\theta )x-\sin (\theta )y,\sin (\theta )x+\cos (\theta )y )$. For any $\theta \in {{\mathrm{SO}}}(2)$, there is a natural action on $C^{\infty }({\mathbb {R}}^2)$ sending the function $\phi \in C^{\infty }({\mathbb {R}}^2)$ to the function $\theta ^*\phi (x,y) := \phi (\cos (\theta )x-\sin (\theta )y,\sin (\theta )x+\cos (\theta )y)$. The corresponding action on vector fields and all other objects on ${\mathbb {R}}^2$ follow naturally. In particular, the left-action on distributions sends $\omega \in {\mathcal {D}}'({\mathbb {R}}^2)$ to the distribution $\theta _* \omega (z) := \omega ( R_\theta \cdot z)$. Again, we can verify that the coadjoint orbit *S* is invariant under this action and that ${{\mathrm{SO}}}(2)$ acts symplectically upon *S* through computations which are analogous to those performed in the previous subsection. Given this symplectic action, we can seek a momentum map, $\mathbf{J}_{\mathrm{ang}}:S \rightarrow \mathfrak {so}(2)^* \equiv {\mathbb {R}}$. Consider an arbitrary element of the Lie-algebra $\xi \in \mathfrak {so}(2) \equiv {\mathbb {R}}$ and use (26) to obtain

$$\begin{aligned} \int \frac{\delta \langle \mathbf{J}_{\mathrm{ang}} (z) , \xi \rangle }{\delta \omega } (z) \, \pounds _{\mathbf {u}}[\omega ] (z) \mathrm{d}z = \int \omega (z) \left( -y\xi v - x\xi u \right) \mathrm{d}z \end{aligned}$$

We can rewrite the right-hand side and integrate by parts to find

$$\begin{aligned} = -\xi \int \omega (z) \pounds _{\mathbf {u}} \left[ \frac{x^2+y^2}{2}\right] dz = \xi \int \pounds _{\mathbf {u}}[\omega ](z) \left( \frac{x^2+y^2}{2} \right) dz. \end{aligned}$$

As the vector $\pounds _{\mathbf {u}}[\omega ] \in T_\omega S$ is arbitrary, we find

$$\begin{aligned} \frac{\delta \langle \mathbf{J}_{\mathrm{ang}} , \xi \rangle }{\delta \omega } = \xi \frac{x^2+y^2}{2} \end{aligned}$$

Integrating by $\omega$, we find

$$\begin{aligned} \mathbf{J}_{\mathrm{ang}}(\omega ) = \frac{1}{2}\int \omega (z) ( x^2 + y^2) dz \in {\mathbb {R}} \equiv \mathfrak {so}(2)^* \end{aligned}$$

If $\omega$ satisfies the MVB ansatz

$$\begin{aligned} \omega = \Gamma _i \delta _{z_i} + \Gamma _i^x \partial _x \delta _{z_i} + \Gamma _i^y \partial _y \delta _{z_i} + \Gamma _i^{xx} \partial _{xx} \delta _{z_i} + \cdots \end{aligned}$$

then

$$\begin{aligned} \mathbf{J}_{\mathrm{ang}}(\omega ) = \frac{\Gamma _i}{2} (x_i^2 + y_i^2) - \Gamma _i^x x_i - \Gamma _i^y y_i + \Gamma _i^{xx} + \Gamma _i^{yy}. \end{aligned}$$

The terms of the MVBs beyond the second order do not influence the angular momentum $\mathbf{J}_{\mathrm{ang}}$.

## Footnotes

1 The choice of *K* should depend on the initial circulation $\omega$, e.g., if the $\omega$ has compact support than any *K* which contains the support of $\omega$ would be a good candidate. Nonetheless, having to choose *K* is a weakness of the given approximation procedure.
